# A lncRNA from an inflammatory bowel disease risk locus maintains intestinal host-commensal homeostasis

**DOI:** 10.1038/s41422-023-00790-7

**Published:** 2023-04-13

**Authors:** Hongdi Ma, Taidou Hu, Wanyin Tao, Jiyu Tong, Zili Han, Dietmar Herndler-Brandstetter, Zheng Wei, Ruize Liu, Tingyue Zhou, Qiuyuan Liu, Xuemei Xu, Kaiguang Zhang, Rongbin Zhou, Judy H. Cho, Hua-Bing Li, Hailiang Huang, Richard A. Flavell, Shu Zhu

**Affiliations:** 1 Department of Digestive Disease, The First Affiliated Hospital of USTC, Division of Life Sciences and Medicine, University of Science and Technology of China, Hefei, Anhui China; 2 Institute of Immunology, the CAS Key Laboratory of Innate Immunity and Chronic Disease, School of Basic Medical Sciences, Division of Life Sciences and Medicine, University of Science and Technology of China, Hefei, Anhui China; 3 Department of Immunobiology, Yale University School of Medicine, New Haven, CT USA; 4 Analytic and Translational Genetics Unit, Massachusetts General Hospital, Harvard Medical School, Boston, MA USA; 5 The Key Laboratory of Digestive Diseases of Anhui Province, Department of Gastroenterology, The First Affiliated Hospital of Anhui Medical University, Hefei, Anhui China; 6 Department of Genetics, Yale School of Medicine, New Haven, CT USA; 7 Shanghai Institute of Immunology, Department of Microbiology and Immunology, Shanghai Jiao Tong University School of Medicine (SJTU-SM), Shanghai, China; 8 Howard Hughes Medical Institute, Yale University School of Medicine, New Haven, CT USA; 9 School of Data Science, University of Science and Technology of China, Hefei, Anhui China; 10 Institute of Health and Medicine, Hefei Comprehensive National Science Center, Hefei, Anhui China

**Keywords:** Autoimmunity, Mechanisms of disease

## Abstract

Inflammatory bowel diseases (IBD) are known to have complex, genetically influenced etiologies, involving dysfunctional interactions between the intestinal immune system and the microbiome. Here, we characterized how the RNA transcript from an IBD-associated long non-coding RNA locus (“***CARINH***-**C**olitis **A**ssociated IRF1 antisense **R**egulator of **In**testinal **H**omeostasis”) protects against IBD. We show that *CARINH* and its neighboring gene coding for the transcription factor IRF1 together form a feedforward loop in host myeloid cells. The loop activation is sustained by microbial factors, and functions to maintain the intestinal host-commensal homeostasis via the induction of the anti-inflammatory factor IL-18BP and anti-microbial factors called guanylate-binding proteins (GBPs). Extending these mechanistic insights back to humans, we demonstrate that the function of the *CARINH/*IRF1 loop is conserved between mice and humans. Genetically, the T allele of rs2188962, the most probable causal variant of IBD within the *CARINH* locus from the human genetics study, impairs the inducible expression of the *CARINH*/IRF1 loop and thus increases genetic predisposition to IBD. Our study thus illustrates how an IBD-associated lncRNA maintains intestinal homeostasis and protects the host against colitis.

## Introduction

Inflammatory Bowel Diseases (IBD) are polygenic chronic inflammatory disorders, and hundreds of IBD-associated genetic loci have been discovered to date.^1^ Interpretation of the molecular and pathogenic mechanisms relating to these loci, however, has been limited to a handful of loci and mostly to coding variants.^2^ Previous studies have implicated long non-coding RNAs (lncRNAs) as regulators of many developmental and physiological processes and as mediators of pathological processes such as autoimmunity.^3^ lncRNAs have important functions in gene regulation, ranging from epigenetic reprogramming to post-transcriptional regulation.^4,5^ It has been reported that ~98% of human genomic mutations occur within non-coding regions.^6^ Moreover, a systematic analysis of NIH Genotype Tissue Expression (GTEx) project data identified 1432 lncRNA gene-trait associations, linking lncRNAs to complex traits and human diseases.^6^

A recent IBD fine-mapping study has pinpointed a genetic association to causal variant candidates in a non-coding region of Chr5: 131.2MB-132.2MB;^2^ it is one of the most significantly associated regions of IBD and is a significantly associated non-coding locus of IBD,^7^ but delineating the specific causal mechanisms and/or genes has proven challenging. Fine-mapping has resolved genetic associations in this region to a 95% credible set of 8 genetic variants (including the most strongly associated variant rs2188962 in this region), all of which are located in the lncRNA locus — *C5orf56*.^2^

In this study, we characterized a lncRNA-*C5orf56* from this IBD-associated region and identified its protective role in IBD. Since this lncRNA interacts with gut microbiota and controls intestinal inflammation, we named it **C**olitis **A**ssociated IRF1 antisense **R**egulator of **In**testinal **H**omeostasis (***CARINH)***. We found that *CARINH* locally promotes *IRF1*’s transcription and forms a *CARINH*/IRF1 regulatory loop in myeloid cells to maintain gut microbiota homeostasis and control intestinal inflammation. Our research reveals the mechanism for the association between non-coding genetic elements and IBD, providing the first insight into the role of an IBD-associated lncRNA in intestinal homeostasis and inflammation.

## Results

### The IBD-associated lncRNA *Carinh* protects against DSS-induced colitis

The *C5orf56* locus, spanning 65.1 kb on human chromosome 5, and its ortholog *Gm12216*, spanning 73.7 kb on mouse chromosome 11 (Supplementary information, Fig. S1a), are highly conserved with 74.2% identity by TransMap alignment (Supplementary information, Fig. S1b). Furthermore, both genes are positioned close to the 3’ end of the innate immunity-related gene *IRF1* (spanning 5.6 kb in human and 7.1 kb in mouse; Supplementary information, Fig. S1a).^8^

We employed the Coding Potential Assessment Tool (CPAT)^9^ to analyze the sequence features of transcript open reading frames (ORFs) to ensure that both human *C5orf56* and its mouse ortholog were transcripts lacking protein-coding potential (Supplementary information, Fig. S1c). We conducted experiments with a mini-circle reporter system containing split green fluorescent protein (GFP) and confirmed that neither the human nor the murine locus had any ribosome-binding capacity (Supplementary information, Fig. S1d).^10,11^ Furthermore, no anti-HA immunoblotting signal was detected upon the expression of an HA-tagged fusion construct for this sequence in HEK293 cells (Supplementary information, Fig. S1e). In addition, the Ribo-seq analysis showed that there are no ribosome binding sites in *Carinh* transcript (Supplementary information, Fig. S1f). Thus, we defined *CARINH* as an IBD-associated and conserved intergenic lncRNA (*hCARINH* for the transcript from *C5orf56* and *mCarinh* for the transcript from *Gm12216*; Supplementary information, Fig. S1a).

**Fig. 1:**
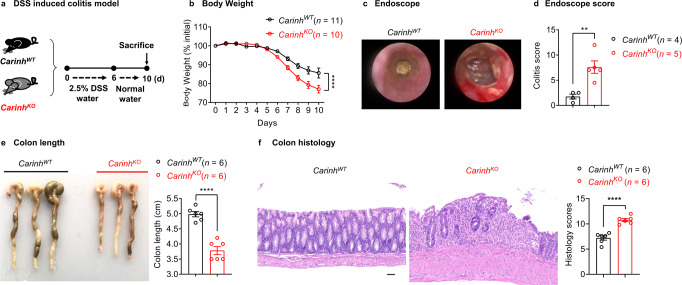
*Carinh* protects against DSS-induced colitis. **a**–**f** DSS (2.5% for 6 days) was administered to induce a colitis model in *Carinh*^*WT*^ and *Carinh*^*KO*^ mice (**a**). Colitis was monitored by body weight loss (**b**), evaluated based on colonoscopy images (**c**) and colitis scores (**d**), colon shortening (**e**), H&E staining (**f**) of colon tissues at day 10 post administration. For H&E staining (**f**): left, representative pictures. Scale bar, 50 μm. Right, quantification of corresponding histology scores. Average scores are from 5 views per mice, *n* = 6 mice per group. Data in **b** are pooled from two independent experiments. Pictures and data in **c**–**f** are representative of three independent experiments. Data are represented as means ± SEM. Body weight changes (**b**) were analyzed by two-way ANOVA. Unpaired two-tailed Student’s *t*-tests were used for the other analyses. **P < 0.01, ****P < 0.0001; ns, not significant.

To study the physiological function of *CARINH* in the intestine and to explore its potential involvement in IBD, we used the CRISPR-Cas9 strategy to generate *Carinh* knockout (*Carinh*^*KO*^) mice on a C57BL/6 genetic background (Supplementary information, Fig. S2a–c). The *Carinh*^*KO*^ mice exhibited normal body weight and their colon tissue showed normal histological features even at 20 weeks old (Supplementary information, Fig. S2d, e). However, upon induction of a classic IBD disease model with dextran sulfate sodium (DSS) (Fig. 1a), the *Carinh*^*KO*^ mice lost significantly more weight (Fig. 1b) and had much more severe bleeding and colitis (assessed via colonoscopy), compared to the DSS-treated wild-type (WT) mice (*Carinh*^*WT*^) (Fig. 1c, d). *Carinh*^*KO*^ mice also exhibited significantly shorter colons (Fig. 1e) and a substantial increase in the extent of inflammatory infiltration and crypt damage (assessed by hematoxylin and eosin (H&E) staining and histology scores), compared to the WT littermates (Fig. 1f). Besides, we also observed evidence of aggravated colitis in the TNBS-induced colitis model involving *Carinh*^*KO*^ mice, but not in their WT littermates (Supplementary information, Fig. S3a–d). These phenotypes imply an increase in the severity of colitis in the colons of the DSS- or TNBS- treated *Carinh*^*KO*^ mice, lending support for the clinical findings from human genetics studies, which have linked *CARINH* with IBD pathogenesis.

### Myeloid-derived *Carinh* protects against DSS-induced colitis

To explore which cell type(s) might be responsible for the effects of *Carinh* in colitis, we first measured the tissue- and cell type-specific expression patterns of *Carinh* in mice. *Carinh* was mainly expressed in lymphoid tissues (the thymus, spleen, mesenteric lymph nodes (MLNs), and the bone marrow (BM)) and mucosal tissue such as the intestine and the lung (Supplementary information, Fig. S4a). In the intestine, *Carinh* was widely expressed in myeloid cells (CD11b^+^), CD4^+^ T cells, CD8^+^ T cells, and EpCAM^+^ intestinal epithelial cells (IECs) (Supplementary information, Fig. S4a). Furthermore, we examined *Carinh* expression in CD11b^+^ myeloid cells sorted from colons of DSS-treated mice and untreated controls. We found that, compared to untreated controls, DSS treatment significantly increased the expression of *Carinh* in CD11b^+^ myeloid cells (Supplementary information, Fig. S4b).

**Fig. 2:**
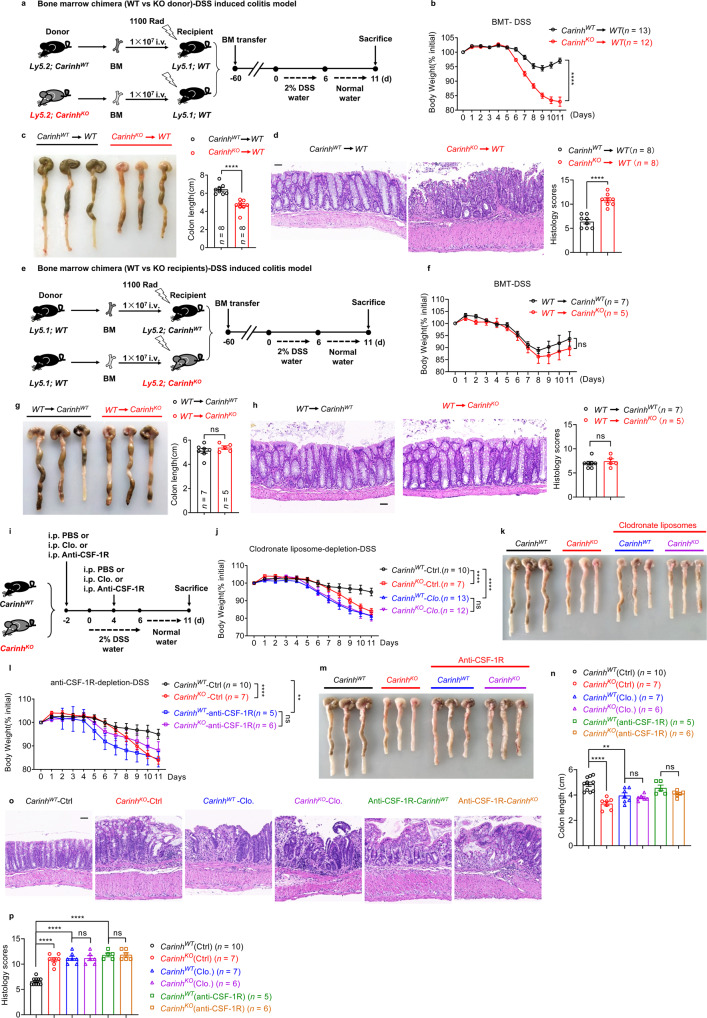
Myeloid-derived *Carinh* protects against DSS-induced colitis. **a**–**d** In BM chimera experiments, BM cells from *Carinh*^*WT*^ and *Carinh*^*KO*^ mice were transplanted into 1100 rad γ-ray irradiated recipient mice. Two months later, the DSS colitis model was induced (**a**). Colitis was monitored by body weight loss (**b**), colon shortening (**c**), and H&E staining of colon tissues (**d**). For H&E staining (**d**): left, representative pictures. Scale bar, 50 μm. Right, quantification of corresponding histology scores. 5 views per mice, 8 mice per group. BMT, bone marrow transplantation. BM cells from congenic mice were transplanted into 1100 rad γ-ray irradiated *Carinh*^*WT*^ and *Carinh*^*KO*^ recipient mice. Two months later, the DSS colitis model was induced (**e**). Colitis was monitored by body weight loss (**f**), colon shortening (**g**), and H&E staining of colon tissues (**h**). For H&E staining (**h**): left, representative pictures. Scale bars, 50 μm. Right, quantification of corresponding histology scores. 5 views per mice, WT to *Carinh*^*WT*^ *n* = 7 mice and WT to *Carinh*^*KO*^ *n* = 5 mice.PBS (Ctrl) or Clodronate liposomes (Clo.) or anti-CSF-1R antibodies were i.p. injected 2 days before (–2d) and 4 days after (+4d) 2% DSS administration (**i**). Colitis was monitored by body weight loss (**j** and **l**), colon shortening (**k** and **m**, representative pictures; **n**, statistics) and H&E staining of colon tissues (**o**, **p**). (**o**) Representative pictures. Scale bars, 50 μm. **p** Quantification of corresponding histology scores. 5 views per mice, 10 *Carinh*^*WT*^ (Ctrl) mice, 7 *Carinh*^*KO*^ (Ctrl) mice, 7 *Carinh*^*WT*^ (Clo.) mice, 6 *Carinh*^*KO*^ (Clo.) mice, 5 *Carinh*^*WT*^ (anti-CSF-1R) mice, and 6 *Carinh*^*KO*^ (anti-CSF-1R) mice. Body weight data in **b**, **f**, **j** and **l** are pooled from two independent experiments. Other data are representative of three independent experiments. Data represent means ± SEM. Body weight changes in **b**, **f**, **j** and **l** were analyzed by two-way ANOVA. Colon length (**n**) and histology scores (**p**) were analyzed by one-way ANOVA. Unpaired two-tailed Student’s *t*-tests were used for other analyses. **P < 0.01, ****P < 0.0001; ns, not significant.

Next, we performed BM chimera experiments, which showed that hematopoietic cells transferred from *Carinh*^*KO*^ mice but not from *Carinh*^*WT*^ mice made recipient mice more susceptible to DSS-induced colitis (Fig. 2a–d). However, when hematopoietic cells were transferred from WT mice, neither the *Carinh*^*KO*^ nor the *Carinh*^*WT*^ recipients exhibited any differences in their colitis (Fig. 2e–h). These results suggest that *Carinh* expression in hematopoietic cells confers the colitis phenotype.

Further, we transferred CD3^+^ T cells isolated from *Carinh*^*KO*^ mice and their WT littermate controls into *Rag1*-deficient mice, and saw that these *Carinh*-deficient T cell-reconstituted mice were not susceptible to DSS-induced colitis (Supplementary information, Fig. S5), suggesting that the expression of *Carinh* in T cells was not required for the DSS phenotype. Additionally, experiments with the CD45RB^hi^ T cells-induced colitis model revealed no difference in the extent of colitis induced by the transfer of *Carinh*^*KO*^ vs *Carinh*^*WT*^ T cells into *Rag1-*deficient mice (Supplementary information, Fig. S6).

Finally, when we used clodronate liposomes to deplete myeloid cells from both *Carinh*^*KO*^ mice and WT littermate controls (Fig. 2i and Supplementary information, Fig. S7), both myeloid cell-depleted *Carinh*^*WT*^ (*Carinh*^*WT*^*-Clo*.) and *Carinh*^*KO*^ (*Carinh*^*KO*^*-Clo*.) mice showed enhanced susceptibility to DSS-induced colitis and had a phenotype similar to that of *Carinh*^*KO*^ mice, in comparison to *Carinh*^*WT*^ mice (Fig. 2i–k and n–p). These results suggest that the expression of *Carinh* in myeloid cells serves a protective role against IBD pathology in the DSS-induced colitis model. Furthermore, to investigate the role of *Carinh* specifically in macrophages during DSS-induced colitis, we used an anti-CSF-1R antibody to inhibit the accumulation and infiltration of macrophages^12–21^ (Fig. 2i and Supplementary information, Fig. S7). When both *Carinh*^*WT*^ *and Carinh*^*KO*^ mice were treated with the anti-CSF-1R antibody, they developed more severe colitis compared to *Carinh*^*WT*^ controls that did not undergo anti-CSF-1R treatment (Fig. 2l–p). These phenotypes are consistent with our findings from the clodronate liposome experiments. Collectively, these results confirm that *Carinh* expression in macrophages serves a protective role against colitis.

### *Carinh* protects against DSS-induced colitis by promoting *Irf1* transcription in myeloid cells

**Fig. 3:**
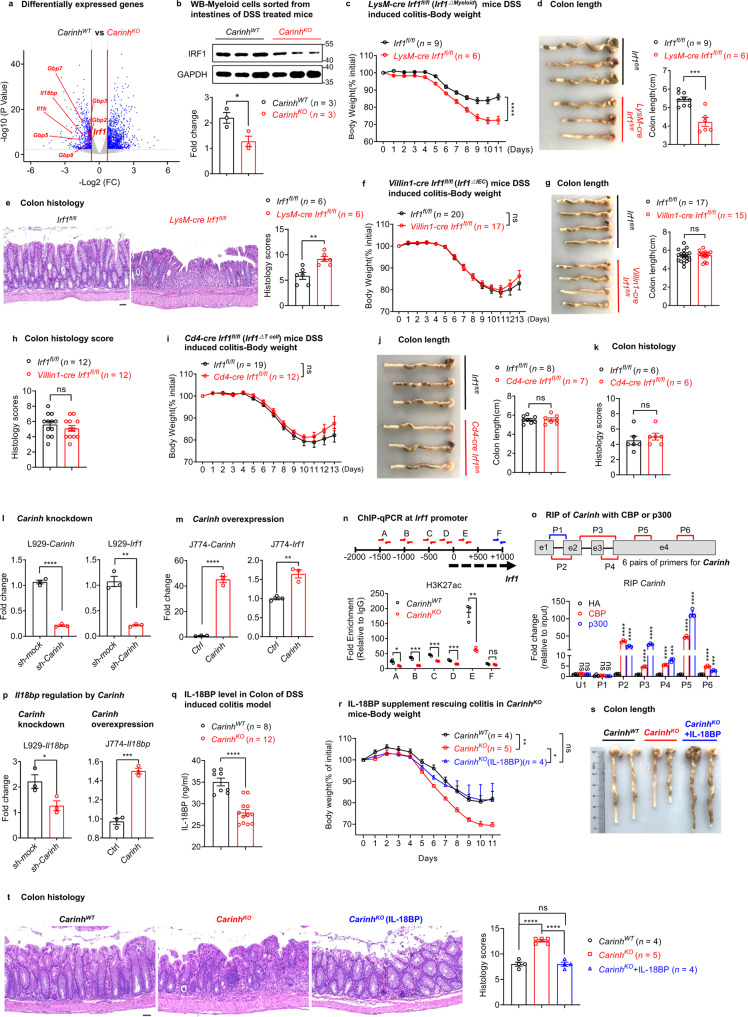
*Carinh* protects against DSS-induced colitis by promoting *Irf1* transcription in myeloid cells. **a** RNA-seq of BMDMs from *Carinh*^*WT*^ and *Carinh*^*KO*^ mice. Volcano plot showing distribution of DEGs between *Carinh*^*WT*^ and *Carinh*^*KO*^ BMDMs. Blue dots (right) and blue dots (left) correspond to genes with significantly increased or decreased expression under each condition (fold changes ratio greater than 1.5 or less than –1.5 with a *P*-value < 0.01). The *x*-axis shows the log_2_ of the fold changes of expression and the *y*-axis shows the *P*-value (–log_10_) for each gene. **b** Western blot assay detecting IRF1 protein expression in CD11b^+^ myeloid cells sorted from intestines of DSS-treated *Carinh*^*WT*^ and *Carinh*^*KO*^ mice (*n* = 3). **c**–**e** DSS-induced colitis model in LysM-cre *Irf1*^△*Myeloid*^ mice. *Irf1*^△*Myeloid*^ mice were administered with 2.5% DSS. Colitis was monitored by body weight loss (**c**), colon shortening (**d**) and H&E staining of colon tissues (**e**). For H&E staining (**e**): left, representative pictures. Scale bars, 50 μm. Right, quantification of corresponding histology scores. 5 views per mice, 6 mice per group. **f**–**h** DSS-induced colitis model in *Irf1*^△*IEC*^ mice. *Irf1*^△*IEC*^ mice were administrated with 2.5% DSS. Colitis was monitored by body weight loss (**f**), colon shortening (**g**) and colon histology scores (**h**). DSS-induced colitis model in *Irf1*^△*T cell*^ mice. *Irf1*^△*T cell*^ mice were administrated with 2.5% DSS. Colitis was monitored by body weight loss (**i**), colon shortening (**j**) and colon histology scores (**k**). qPCR analyses of *Carinh* and *Irf1* mRNA expression in L929 cells transduced with *Carinh*-specific shRNAs or scramble control (**l**), or in murine macrophage cell lines (J774) transduced with *Carinh* plasmid or empty vector (**m**). *n* = 3 per group. **n** ChIP-qPCR for H3K27ac at the *Irf1* promoter in BM cells. A–E represent selected qPCR primers in the indicated locations at *Irf1* promoter; F represents qPCR primer in non-relevant region. Data are presented as enrichment fold over the IgG control. *n* = 3 per group. **o** Co-immunoprecipitation analysis between *Carinh* RNA and H3K27ac modifier p300/CBP. Protein extracts from HEK293 cells transduced with HA-tagged p300 or HA-tagged CBP and *Carinh* RNA, were immunoprecipitated with HA antibodies. RNAs binding to p300 or CBP were assayed by qPCR. P1 to P6 represent selected qPCR primers in *Carinh* region. U1 represents a non-relevant control. Data are presented as relative expression of input. *n* = 3 per group. **p** qPCR analyses of *Il18bp* expression in L929 cells transduced with *Carinh*-specific shRNAs or scramble control (left), or in murine macrophage cell lines (J774) transduced with *Carinh* plasmid or empty vector (right). *n* = 3 per group. **q** ELISA detection of IL-18BP levels in colon homogenates of DSS-induced *Carinh*^*WT*^ (*n* = 8) and *Carinh*^*KO*^ (*n* = 12) mice. IL-18BP supplement in DSS-induced colitis model. *Carinh*^*KO*^ mice were treated with 2.5% DSS and intraperitoneally (i.p.) injected with or without IL-18BP. Colitis was monitored by body weight loss (**r**), colon shortening (**s**) and H&E staining of colon tissues (**t**). For H&E staining (**t**): left, representative pictures. Scale bars, 50 μm. Right, quantification of corresponding histology scores. 5 views per mice, *Carinh*^*WT*^ mice *n* = 4, *Carinh*^*KO*^ mice *n* = 5, *Carinh*^*KO*^ mice with IL-18BP treatment *n* = 4. Data in **d**, **e**, **g**, **j**, **s** and **t** are representative of 3 independent experiments. Body weight in **f** and **i** are pooled from 3 independent experiments. Data in **q** are pooled from 2 independent experiments. Data from in vitro experiments are representative of at least three independent experiments. Data represent means ± SEM. Body weight changes in **c**, **f**, **i** and **r** were analyzed by two-way ANOVA. Histology scores (**t**) were analyzed by one-way ANOVA. Unpaired two-tailed Student’s *t*-tests were used for the other statistical analyses. *P < 0.05, **P < 0.01, ***P < 0.001, ****P < 0.0001; ns, not significant.

We next sought to determine how *Carinh* expression in myeloid cells protects against IBD. We first isolated and differentiated BM-derived macrophages (BMDMs) from *Carinh*^*KO*^ mice and WT littermates and conducted RNA sequencing (RNA-seq). Intriguingly, the expression of *Carinh*’s neighboring gene *Irf1*, as well as its transcriptional targets (e.g., *Il18bp* and *Gbps*) were among the down-regulated differentially expressed genes (DEGs) in *Carinh*^*KO*^ BMDMs (Fig. 3a). We also confirmed the down-regulation of the *Irf1* transcript by real-time quantitative PCR (RT-qPCR) in the BM of *Carinh*^*KO*^ mice (Supplementary information, Fig. S8a).

It has been reported that many lncRNAs exert localized effects and influence the expression of neighboring genes.^22^ In addition, it has been shown that lncRNAs are uniquely poised to regulate their genomic neighborhoods *in* *cis*.^4^ Since both human *CARINH* and mouse *Carinh* loci are positioned close to the 3′ end of *IRF1/Irf1* (spanning 5.6 kb in human and 7.1 kb in mouse) (Supplementary information, Fig. S1a), we proposed that *Carinh* regulates its neighboring gene *Irf1* to exert a protective effect against colitis. Indeed, a genome-wide association study (GWAS)^23^ and a more recent Crohn’s Disease exome sequencing study involving over 100,000 subjects (*P* = 8 × 10^−17^; https://github.com/yorkklause/Crohn-s-Disease-WES-meta), have shown that *IRF1* is also an IBD-associated gene. Moreover, we found that *Carinh* deficiency significantly decreased the expression of IRF1 at protein level in the intestinal myeloid cells under the physiological (without DSS induction, Supplementary information, Fig. S8b) and pathological (with DSS induction, Fig. 3b) conditions. Thus, we speculated whether *Carinh* protects against DSS-induced colitis through the regulation of IRF1. We first generated *Irf1* knockout (*Irf1*^*KO*^) mice and observed exacerbated DSS-induced colitis phenotypes compared to their WT littermates (*Irf1*^*WT*^) (Supplementary information, Fig. S8c–g), providing support for previous studies.^24,25^ However, as a transcriptional factor, IRF1 regulates genes with multiple functions in different cell types, in response to stimuli that vary under different conditions.^26^ Therefore, to validate the cell-specific role of IRF1, we crossed newly generated, CRISPR-edited, *Irf1*-floxed mice with different cell type-specific Cre lines, including Villin1-cre (*Irf1*^△*IEC*^), Cd4-cre (*Irf1*^△*T cell*^), and LysM-cre (*Irf1*^△*Myeloid*^) (Supplementary information, Fig. S8h). The *Irf1*^△*Myeloid*^ mice (Fig. 3c–e) but not the *Irf1*^△*IEC*^ (Fig. 3f–h and Supplementary information, Fig. S8i) or the *Irf1*^△*T cell*^ (Fig. 3i–k and Supplementary information, Fig. S8j) mice were susceptible to DSS-induced colitis. Beyond supporting our results from the aforementioned clodronate liposome or anti-CSF-1R experiments, showing myeloid-specific contributions of *Carinh* to colitis, these findings suggest that myeloid-specific disruption of both *Carinh* and *Irf1* causes IBD pathology.

To understand how *Carinh* regulates *Irf1*, we first investigated the mechanism in vitro. Knocking down *Carinh* caused a significant decrease in *Irf1* mRNA levels in L929 cells (Fig. 3l), whereas we observed significantly increased *Irf1* mRNA levels upon *Carinh* overexpression (Fig. 3m), indicative of transcriptional regulation. Conversely, we detected no differences in the expression of other *Irf* members (including *Irf3, Irf7*, and *Irf8*) between *Carinh*^*KO*^ and *Carinh*^*WT*^ cells, thereby excluding a non-specific regulatory impact of *Carinh* on interferon signaling pathway activity (Supplementary information, Fig. S9a). In addition, knocking out *Carinh* did not affect the expression of another neighboring gene *Slc22a5* (Supplementary information, Fig. S9b), which further emphasized the specificity of *Carinh*’s regulation of *Irf1*.

We further investigated the mechanism by which *Carinh* RNA promotes *Irf1* transcription. Genes that require tight transcriptional regulation but must retain the capacity to be rapidly regulated on a frequent basis often feature histone modifications at their promoters.^27,28^ During the transcriptional activation of a gene, the extent of histone modifications that are associated with transcriptional activation (e.g., H3K4me3 and H3K27ac) increases, whereas, during transcriptional suppression, increased levels of H3K27me3 repressive histone modifications are observed.^27,28^ A number of lncRNA transcripts have been shown to regulate the transcription of their neighboring genes;^29–32^ mechanistically, these lncRNA transcripts function to increase the target-gene occupancy rates of epigenetic mark writer enzymes like p300/CBP (for H3K27ac) and Wdr5 complex subunits (for H3K4me3).^29,33,34^ ENCODE (Encyclopedia of DNA Elements) project data showed strong evidence of H3K4me3 and H3K27ac modifications around the transcriptional start site (TSS) of *Irf1* in both mouse BMDMs and intestinal tissues (Supplementary information, Fig. S10a, b), while H3K27ac modification was not found at the locus of *Slc22a5* (another neighboring gene not regulated by *Carinh*) in murine BMDMs (Supplementary information, Fig. S10c). Notably, by analyzing public chromatic immunoprecipitation sequencing (ChIP-seq) datasets using the CistromeDB Toolkit, we also confirmed that H3K27ac was the most likely epigenetic modification involved in the regulation of *Irf1* expression and the surrounding chromosomal region (Supplementary information, Fig. S10d).

We therefore hypothesized that *Carinh* RNA may increase the deposition of epigenetic modifications by recruiting their writers to the *Irf1* promoter, thereby promoting *Irf1* transcription. Pursuing this notion, we performed chromatin immunoprecipitation followed by quantitative PCR (ChIP-qPCR) analysis of BM cells derived from *Carinh*^*KO*^ and WT littermate mice, using antibodies against H3K27ac or H3K4me3. In line with our earlier results, showing reduced levels of *Irf1* transcription in the BM of *Carinh*^*KO*^ mice (Supplementary information, Fig. S8a), our ChIP-qPCR data showed a significant reduction in H3K27ac levels at the *Irf1* promoter of BM cells from *Carinh*^*KO*^ mice compare to that from *Carinh*^*WT*^ mice (Fig. 3n), while there was no difference in H3K4me3 levels at the *Irf1* promoter between *Carinh*^*WT*^ and *Carinh*^*KO*^ cells (Supplementary information, Fig. S10e). As a control, there was also no difference in H3K9ac levels at the *Irf1* promoter between *Carinh*^*WT*^ and *Carinh*^*KO*^ cells (Supplementary information, Fig. S10e). In addition, our H3K27ac ChIP-qPCR data also showed that there is no difference of H3K27ac levels at the promoter of *Irf3*, *Irf7*, *Irf8* and *Slc22a5* (Supplementary information, Fig. S10f), which is consistent with the results in Supplementary information, Fig. S9a, b that *Carinh* did not regulate other members of *Irfs* as well as another neighboring gene *Slc22a5*. These data further confirmed the specific regulation of *Irf1* by *Carinh*. We next asked whether *Carinh* RNA could physically interact with p300/CBP, the key epigenetic writer proteins of the H3K27ac modification. RNA-binding protein immunoprecipitation (RIP) experiments in HEK293 cells, overexpressing recombinant epigenetic writer proteins and *Carinh* RNA, revealed a strong interaction between the *Carinh* transcript and p300 or CBP (Fig. 3o). Also, CBP RIP-seq data analyzed from ENCODE project showed strong signals at *CARINH* exons (Supplementary information, Fig. S10g), suggesting the direct binding of CBP to *CARINH* RNA. In addition, by analyzing p300 and CBP ChIP-seq data from public datasets,^35,36^ we found that both p300 and CBP could bind to the promoter region of *Irf1* (Supplementary information, Fig. S10h). These results demonstrate that *Carinh* RNA promotes *Irf1* expression by interacting with p300/CBP to promote the deposition of activating histone marks H3K27ac at the *Irf1* locus.

Moreover, we identified three annotated isoforms of *Carinh* in myeloid cells sorted from mouse colons (Supplementary information, Fig. S11a). Using RIP-qPCR, we found that isoform *Gm12216-201* (the longest isoform) displayed the strongest interactions with p300 or CBP (Supplementary information, Fig. S11b). In accordance, we observed that the isoform *Gm12216-201* was responsible for the strongest induction of *Irf1* transcription in a macrophage cell line RAW264.7 (Supplementary information, Fig. S11c). Further, to investigate the binding regions of *Carinh* RNA to CBP and p300, we used RIP experiments which showed that full-length *Carinh* RNA but not the RNA transcripts encoded by *Carinh* Exon 1–2 bind to CBP or p300 (Supplementary information, Fig. S11d), indicating the RNA transcript encoded by *Carinh* Exon 3–4 or the complete *Carinh* RNA structure is required for the interaction with these epigenetics modulators.

We further investigated the mechanism by which myeloid *Carinh* promotes *Irf1* transcription to protect against DSS-induced colitis. Recalling our RNA-seq data in *Carinh*^*WT*^ and *Carinh*^*KO*^ BMDMs, Gene Ontology (GO) analysis of the DEGs revealed that the down-regulated genes of *Carinh*^*KO*^ macrophages were enriched for functional annotations related to the regulation of external stimulus response pathways (specifically inflammatory response, defense response, response to molecules of bacterial origin, and response to LPS; Supplementary information, Fig. S12a). We noticed that *Irf1* and its known transcriptional targets including *Gbps, Il18bp*, and a well-known inflammatory cytokine *Il1b* are in the pathway (Fig. 3a), which have been reported to be involved in the inflammatory response and defense response. We tested the potential involvement of these genes for their potential role in the pathogenesis of IBD, including IL-18BP,^25,37^ IL-1β^38^ and *Gbps*.^39^ We also tested another IRF1 down-stream effector IL-15^40^ as it has been reported to maintain the population of intra-epithelial lymphocytes.

We analyzed the protein level of IL-1β in the homogenates of colon from DSS-induced *Carinh*^*WT*^ and *Carinh*^*KO*^ mice. Results from ELISA experiment showed that there is no difference in the level of IL-1β secreted into the colon during DSS induction between *Carinh*^*WT*^ and *Carinh*^*KO*^ mice (Supplementary information, Fig. S12b), indicating that IL-1β might not be the effector that mediated the *Carinh* deficiency-caused colitis phenotype.

We also examined the *Il15* expression in BM cells or intestinal tissues from *Carinh*^*WT*^ and *Carinh*^*KO*^ mice. The expression levels of *Il15* showed no difference between *Carinh*^*WT*^ and *Carinh*^*KO*^ mice (Supplementary information, Fig. S12c). Furthermore, since IL-15 is essential to maintain homeostatic proliferation of lymphocytes, we compared the number of mononuclear cells (MNCs) in spleen, BM, MLNs, as well as the number of ileac lamina propria lymphocytes (I-LPL), ileac intraepithelial lymphocytes (I-IEL), colonic lamina propria lymphocytes (C-LPL) and colonic intraepithelial lymphocytes (C-IEL) between *Carinh*^*WT*^ and *Carinh*^*KO*^ mice. There is no difference in the number of these cells between *Carinh*^*WT*^ and *Carinh*^*KO*^ mice (Supplementary information, Fig. S12d). These results suggested that IL-15 is not responsible for the *Carinh* deficiency-mediated colitis phenotype.

The IL-18BP promoter contains an IRF1-binding site;^41^ IRF1 KO mice have undetectable serum IL-18BP levels and strongly reduced *Il18bp* mRNA level in the liver and spleen.^41,42^ Our data also supported the regulation of *Carinh* and IRF1 to *Il18bp* in macrophages. The induction of *Il18bp* mRNA is significantly reduced in *Carinh*-deficient BMDMs in response to LPS stimulation with time-course (Supplementary information, Fig. S12e). Moreover, *Il18bp* mRNA could not be induced by LPS in *Irf1* KO BMDMs, suggesting the strong regulation of *Il18bp* by IRF1(Supplementary information, Fig. S12e).

Our previous study reported that depletion of IL-18BP resulted in increased bioavailability of IL-18, which exacerbated colitis severity.^37^ We thus speculated that the development of severe colitis in the *Carinh*^*KO*^ mice may result from a reduction in IL-18BP levels. We first examined the regulatory relationship between *Carinh* and *Il18bp*. Compared to control materials, the *Il18bp* mRNA level was significantly reduced in a murine cell line with shRNA-mediated knockdown of *Carinh* (Fig. 3p). Conversely, overexpression of *Carinh* in a murine macrophage cell line caused a significant increase in the *Il18bp* mRNA levels (Fig. 3p). By complementing *Carinh* or *Irf1* into *Carinh*^*KO*^ BMDMs, we found that reconstitution of either *Carinh* or *Irf1* restored the decreased expression of *Carinh*, *Irf1* and *Il18bp* caused by the deficiency of *Carinh* (Supplementary information, Fig. S12f). These data further confirmed the RNA-based regulation of *Irf1* and *Il18bp* by *Carinh*.

Moreover, analysis of colon homogenates from a DSS-induced colitis model mice showed that the protein levels of IL-18BP were significantly reduced in *Carinh*^*KO*^ mice, compared with their WT littermates (Fig. 3q). These results indicate that *Carinh* functions as a positive regulator of *Il18bp* and support our hypothesis that the aggravated colitis observed in the *Carinh*^*KO*^ mice could result from insufficient IL-18BP levels. Providing further support for this, we found that administering recombinant IL-18BP to *Carinh*^*KO*^ mice reversed the severe colitis phenotype of *Carinh*^*KO*^ mice, as evidenced by our histological observations and the ultimate restoration of body weight and colon length (Fig. 3r–t).

### Intestinal microbiota sustains the expression of a *Carinh/*IRF1 feedforward loop to maintain microbial homeostasis

**Fig. 4:**
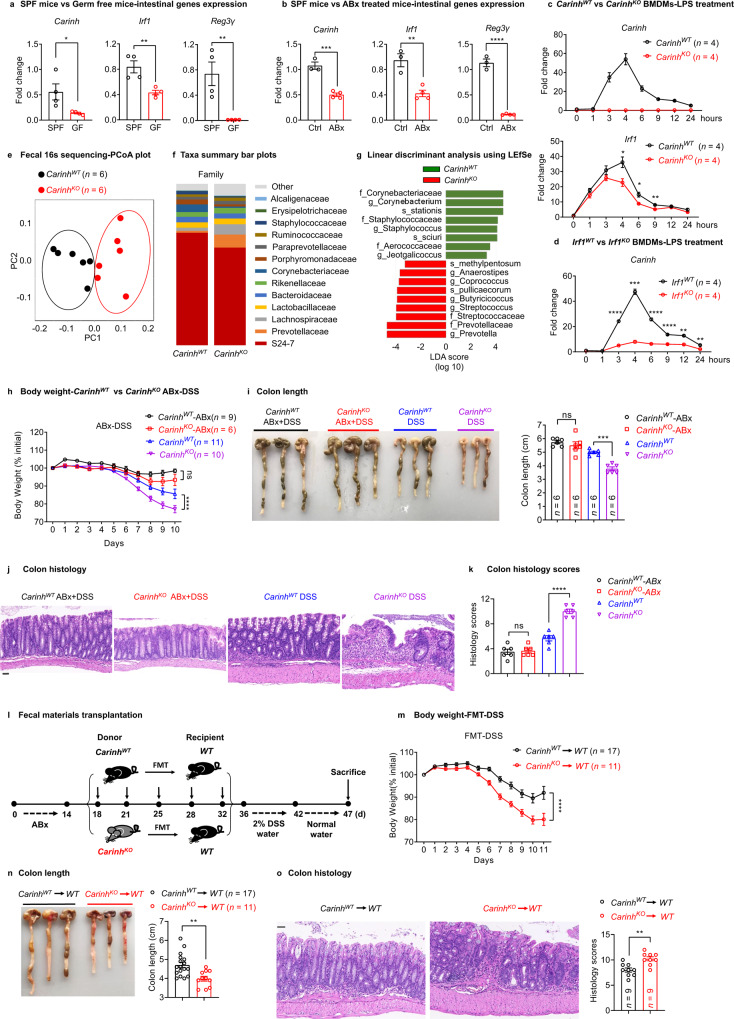
Intestinal microbiota sustains the expression of a *Carinh/*IRF1 feedforward loop to maintain microbial homeostasis. **a**, **b** qPCR analysis of *Carinh, Irf1* and *Reg3γ* mRNA expression in the intestine from SPF and GF mice (**a**) (*n* = 4 mice per group), or from ABx-treated and untreated mice (**b**) (Ctrl *n* = 3 mice, ABx *n* = 4 mice). **c**, **d** qPCR analysis of *Carinh* and *Irf1* mRNA levels in *Carinh*^*KO*^ BMDMs (**c**), *Irf1*^*KO*^ BMDMs (**d**), and their littermate controls in response to LPS stimulation with time-course. *n* = 4 per group. **e** Fecal 16S rDNA sequencing results for *Carinh*^*KO*^ mice and their littermates. PCoA plot generated from unweighted UniFrac distance matrix displaying the distinct clustering pattern for the intestinal bacteria communities of *Carinh*^*KO*^ mice and their littermates. **f** Relative abundance of taxonomic groups averaged across *Carinh*^*KO*^ mice and their littermates at the family level. **g** Composition differences of the intestinal microbiota in *Carinh*^*KO*^ mice and their littermates, determined by linear discriminant analysis using LEfSe. **h**–**k** DSS induced colitis model in ABx-treated or untreated *Carinh*^*KO*^ mice and their littermates. Colitis was monitored by body weight loss (**h**), colon shortening (**i**), and H&E staining of colon tissues (**j**, **k**). **j** Representative pictures. Scale bars, 50 μm. **k** Quantification of corresponding histology scores. 5 views per mice, 6 mice per group. **l** Fecal microbiota from *Carinh*^*WT*^ and *Carinh*^*KO*^ mice were transferred into ABx-treated WT recipients. And then colitis was induced by DSS in the FMT recipient mice. **m**–**o** Colitis was monitored by body weight loss (**m**), colon shortening (**n**), and H&E staining of colon tissues (**o**). For H&E staining (**o**): left, representative pictures. Scale bars, 50 μm. Right, quantification of corresponding histology scores. 5 views per mice, 9 mice per group. 6 pairs of *Carinh*^*WT*^ and *Carinh*^*KO*^ mice were used in 16S rDNA sequencing experiments shown in **e**–**g** and were representative of two independent 16S rDNA sequencing experiments. Data in **i**, **j**, **n** and **o** are representatives of three independent experiments. Body weight data in **h** and **m** are pooled from two independent experiments. Data in **a–d** are representative of at least three independent experiments. Data represent means ± SEM. Body weight changes (**h** and **m**) were analyzed by two-way ANOVA. Colon length (**i**) and histology scores (**k**) were analyzed by one-way ANOVA. Unpaired two-tailed Student’s *t-*tests were used for other statistical analyses. *P < 0.05, **P < 0.01, ***P < 0.001, ****P < 0.0001; ns, not significant.

Since we have shown that *Carinh* positively regulates IRF1 and IL-18BP to control intestinal inflammation, we next wanted to explore whether any environmental factors in the intestine regulate the *Carinh/*IRF1/IL-18BP axis. Dysfunctional interactions between the intestinal immune system and gut microbiota are a major cause of IBD. Considering that the gut lumen is known to host a dense population of commensal bacteria, we first investigated the interactions between *Carinh* and gut microbiota. We measured the expression levels of *Carinh*, *Irf1*, and *Il18bp* in Specific Pathogen Free (SPF) mice, as well as in germ-free (GF) mice or antibiotic-treated (ABx) mice, both of which are deficient in commensal bacteria. Intriguingly, the transcription levels of *Carinh*, *Irf1*, and *Il18bp* were significantly reduced in the intestinal tissues of GF (Fig. 4a and Supplementary information, Fig. S12g) and ABx mice (Fig. 4b and Supplementary information, Fig. S12h), compared to SPF control mice. We also found that the secreted IL-18BP levels were significantly lower in the intestinal homogenates of GF mice than those of SPF mice (Supplementary information, Fig. S12i). In addition, analysis from a single cell dataset of mouse intestinal immune cells derived from the GEO database also showed the reduced expression of *Carinh*, *Irf1*, and *Il18bp* in intestinal CD11b^+^ myeloid cells in GF and ABx mice, compared to the SPF controls (Supplementary information, Fig. S12j). These results support the notion that intestinal bacteria sustain the expression of *Carinh* and *Carinh*-regulated genes.

We next used BMDMs to study how bacterial stimuli impact on the *Carinh*/*Irf1/Il18bp* axis in vitro. We used the bacterial cell wall component LPS, a well-known microbial signal, to mimic bacterial stimulation.^43^ In line with previous studies,^44,45^ *Irf1* mRNA levels were induced within 1 h and peaked on 4 h upon LPS exposure (Fig. 4c). Meanwhile, *Carinh* RNA expression was induced after 3 h and peaked on 4 h following LPS treatment (Fig. 4c, d), meaning that *Irf1* expression was induced ahead of *Carinh*, and *Carinh*/*Irf1* module may work as an immune response gene unit. Interestingly, the *Carinh*^*KO*^ mice exhibited weaker induction of *Irf1* transcription as well as IRF1 protein expression upon LPS treatment (Fig. 4c and Supplementary information, Fig. S12k). Similarly, a less marked increase in *Carinh* RNA levels was observed following LPS stimulation of *Irf1*^*KO*^ BMDMs (Fig. 4d). In addition, we observed a substantial decrease in *Carinh* RNA levels in multiple *Irf1*^*KO*^ murine tissues (Supplementary information, Fig. S13a). *Irf1* overexpression in murine cell lines, however, significantly increased *Carinh* RNA levels (Supplementary information, Fig. S13b). These findings clearly suggest that *Irf1* positively regulates *Carinh* transcription. Supporting this, there are predicted IRF1-binding sites in the 5’ sequence near the TSS of the *Carinh* locus (Supplementary information, Fig. S13c). ChIP analysis of BMDMs using an antibody against IRF1 showed that IRF1 directly binds to the *Carinh* promoter and promotes its transcription (Supplementary information, Fig. S13d). Thus, the colonization of intestinal microbiota and a microbial signal can sustain the expression of *Carinh* and IRF1, and they interact via a positive feedforward loop.

We next assessed if the microbiota-sustained *Carinh*/IRF1 feedforward loop can impact bacterial composition in the intestine. We performed 16S sequencing to analyze the composition of bacterial taxa in fecal samples from *Carinh*^*KO*^ and *Irf1*^*KO*^ mice, and their corresponding WT littermates (separated at the time of weaning). Compared to their respective littermate controls, there were substantial changes in the microbiota composition of both *Carinh*^*KO*^ (Fig. 4e–g) and *Irf1*^*KO*^ mice (Supplementary information, Fig. S14a–c), with a signature of the enrichment of the inflammatory commensal *Prevotellaceae*.^46–51^ To test whether this alteration of gut microbiota in the *Carinh*^*KO*^ and *Irf1*^*KO*^ mice exacerbates intestinal inflammation, we first treated mice with antibiotics (ABx) and then induced colitis using the DSS model. Notably, the increase in DSS-induced colitis severity observed in the *Carinh*^*KO*^ (Fig. 4h–k) and *Irf1*^*KO*^ (Supplementary information, Fig. S14d–f) mice was abolished upon the elimination of intestinal bacteria via antibiotic treatment. We further performed the fecal materials transplantation (FMT) experiments to test microbial contribution to the induction of colitis (Fig. 4l). We transferred gut microbiota from *Carinh*^*KO*^ mice or *Carinh*^*WT*^ mice to the ABx-treated WT recipients and found that FMT from *Carinh*^*KO*^ mice aggravated the DSS-induced colitis in the recipients, compared to the recipients of FMT from *Carinh*^*WT*^ controls (Fig. 4l–o). These findings suggest that *Carinh* deficiency perturbed the gut microbiome and this change in microbiota composition contributed to the aggravated colitis observed in *Carinh*^*KO*^ mice. Thus, microbe signal-sensitive *Carinh*/IRF1 positive feedforward loop maintains a healthy distribution of intestinal microbiota.

We next explored the question of how *Carinh* affects intestinal microbiota composition. Recalling our RNA-seq data in Fig. 3a, many known transcriptional targets of IRF1 (e.g., *Gbp2, Gbp3, Gbp5, Gbp7*, and *Gbp8*)^26,52,53^ were among the down-regulated DEGs in *Carinh*^*KO*^ macrophages (Fig. 3a and Supplementary information, Fig. S15a). Notably, genes encoding five members of the GBP family, which were reported to have important functions in anti-microbial defense and are known targets of IRF1,^52–55^ were down-regulated in *Carinh*^*KO*^ macrophages compared to *Carinh*^*WT*^ macrophages (Supplementary information, Fig. S15a). Consistent with the *Carinh* and *Irf1* expression trends (Fig. 4a, b), GF or ABx treatment conditions reduced the expression of *Gbps* in mouse intestines (Supplementary information, Fig. S15b, c) and in intestinal myeloid cells (Supplementary information, Fig. S15d), suggesting that GBPs are induced by microbes. During microbial signal LPS exposure, however, the induction of *Gbps* was impaired in the macrophages from *Carinh*^*KO*^ and *Irf1*^*KO*^ mice, compared to that from the littermate control mice (Supplementary information, Fig. S15e, f). These results indicate that the *Carinh*/IRF1 feedforward loop regulates the defense response to intestinal stimuli and maintains intestinal homeostasis, potentially through the modulation of anti-microbial factors — GBPs.

### *CARINH* is functionally conserved as a regulator of inflammation in human

Having demonstrated the existence of a bacteria-sustained *Carinh/Irf1* regulatory loop that dictates the species composition of the gut microbiome and regulates intestinal inflammation in mice (and recalling the genetic association between IBD and the *C5orf56* locus in humans), we asked whether the expression pattern and the function of this loop are conserved in humans. Analysis of GTEx (NIH Genotype-Tissue Expression) project data showed that *CARINH* levels were relatively high in human Epstein-Barr virus (EBV)-transformed lymphocytes, as well as the spleen, the thyroid, the small intestine, and the colon (*P* = 0.002 for *C5orf56*, two-sided Wilcoxon rank-sum test) (Supplementary information, Fig. S4c). This expression pattern is consistent with the high expression of *Carinh* observed in the lymphoid and mucosal tissues of mice (Supplementary information, Fig. S4a).

**Fig. 5:**
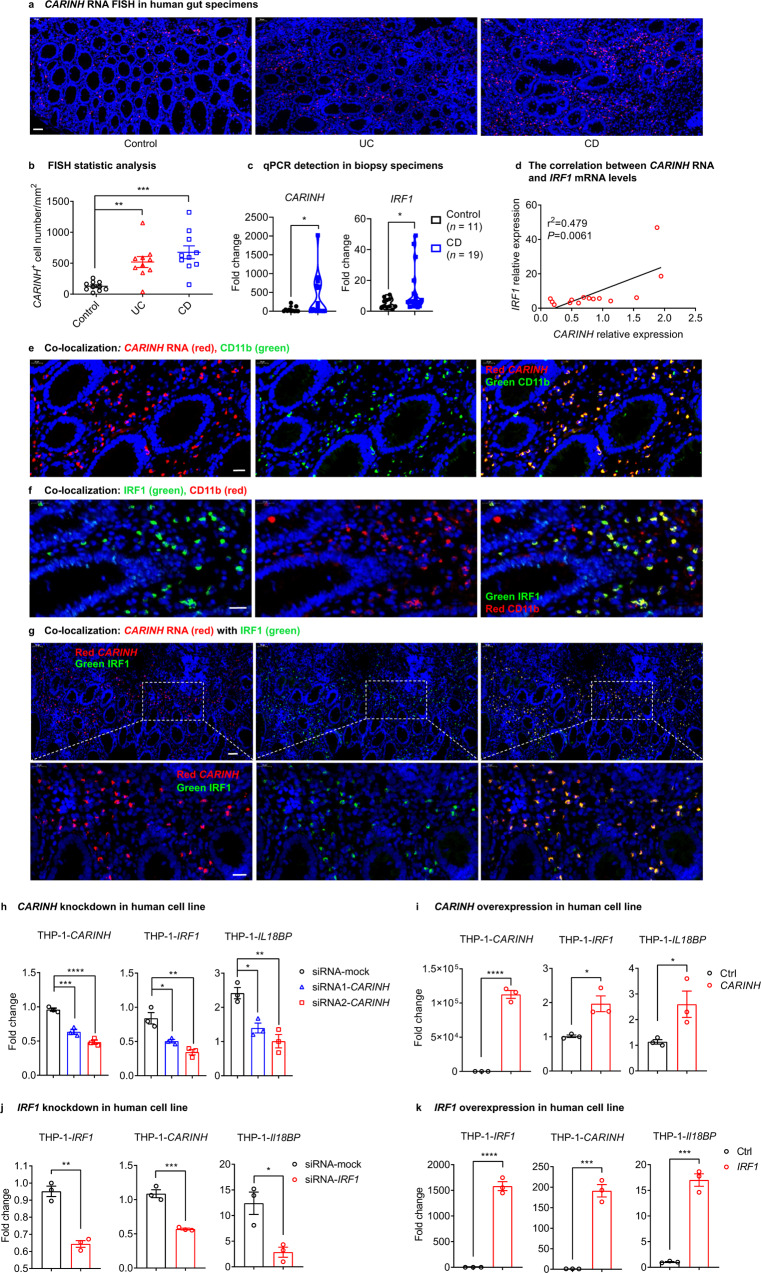
*CARINH* is functionally conserved as a regulator of inflammation in human. **a** *CARINH* RNA expression in human gut specimens from control individuals (left, *n* = 10), UC patients (middle, *n* = 10), and CD patients (right, *n* = 10), assessed by RNA FISH. Representative pictures are shown. Scale bar, 50 μm. **b** Quantification of the FISH results from **a**, shown as the *CARINH* RNA-positive cell count per mm^2^. *n* = 10 per group. **c** *CARINH* and *IRF1* mRNA levels are detected by qPCR in terminal ileum biopsy specimens. Control, *n* = 11; CD, *n* = 19. **d** *CARINH* and *IRF1* mRNA levels are detected by qPCR in PBMCs of IBD patients. The correlation was analyzed between *CARINH* RNA and *IRF1* mRNA levels. **e** FISH and immunofluorescence staining, showing the co-localization of *CARINH* RNA (red) with CD11b (green). Yellow indicates co-localization. DAPI (blue) stains cell nuclei. Scale bars, 20 μm. **f** Immunofluorescence staining showing the co-localization of IRF1 (green) with CD11b (red). Yellow indicates co-localization. DAPI (blue) stains cell nuclei. Scale bars, 20 μm. **g** FISH and immunofluorescent staining showing the co-localization of *CARINH* RNA (red) with IRF1 (green). Yellow indicates co-localization. DAPI (blue) stains the nuclear of cell. The bottom panel of images are magnified views of the areas indicated by white line boxes above. Up panel scale bar, 50 μm. Bottom panel scale bar, 20 μm. **h**, **i** qPCR analyses of human *CARINH, IRF1* and *IL18BP* mRNA expression in THP-1 cells transduced with human *CARINH*-specific siRNAs or scramble control (**h**), and with human *CARINH-*expressing vector or empty vector (**i**). *n* = 3 per group. **j**, **k** qPCR analyses of human *IRF1, CARINH* and *IL18BP* mRNA expression in THP-1 cells transduced with human *IRF1*-specific siRNAs or scramble control (**j**), and with human *IRF1-*expressing vector or empty vector (**k**). *n* = 3 per group. The data in **h**–**k** are representative of at least three independent experiments. Data represent means ± SEM. Data in **b** and **h** were analyzed by one-way ANOVA. Unpaired two-tailed Student’s *t*-tests were used for other statistical analyses. *P < 0.05, **P < 0.01, ***P < 0.001, ****P < 0.0001.

We next used RNA fluorescence in situ hybridization (FISH) to evaluate *CARINH* RNA in colonic resection specimens from a cohort of 20 patients with IBD (10 with Crohn’s disease (CD) and 10 with ulcerative colitis (UC); Supplementary information, Table S2). Of note, none of these patients harbored any IBD-associated mutations in the *CARINH* and *IRF1* loci. Strikingly, the expression of *CARINH* RNA was increased in gut specimens from both CD and UC patients, compared to control biopsy tissues, with an especially pronounced increase observed in the CD patients (Fig. 5a, b). qPCR detection of terminal ileum biopsy specimens from another cohort of 19 CD patients and 10 healthy controls further confirmed the observation that *CARINH* and *IRF1* expression levels were increased in CD patients compared with the control specimens (Fig. 5c). Also, we found that *CARINH* RNA levels in peripheral blood mononuclear cell (PBMC) positively correlate with *IRF1* mRNA levels (Fig. 5d). Previous studies have reported that IRF1 protein levels were upregulated in both human IBD specimens and in the colons of mice with DSS-induced colitis.^24,56^ Moreover, a strong association between IRF1 levels and clinical indices of disease activity has been observed in patients with IBD.^56^ Thus, we argued for the existence of a conserved *CARINH*/IRF1 regulatory loop expression pattern in IBD specimens.

Indeed, combining FISH and immunofluorescence staining techniques confirmed that *CARINH* RNA and IRF1 were localized to human myeloid cells (Fig. 5e, f), consistent with our findings in mice. Moreover, our finding that the expression pattern of *CARINH* RNA in gut specimens from patients with IBD corresponds with IRF1 protein accumulation (Fig. 5g), supports the notion that the *CARINH*/IRF1 feedforward loop exists in humans.

Therefore, we next set out to examine the functional conservation of the *CARINH/IRF1* loop in humans. We observed that the knockdown of *CARINH* in human monocyte THP-1 cells caused a significant reduction in the levels of both *IRF1* and *IL18BP* mRNA (Fig. 5h). In contrast, the overexpression of *CARINH* RNA in THP-1 cells led to the significant increase in the levels of both *IRF1* and *IL18BP* (Fig. 5i). We also found the same trends for *CARINH* and *IL18BP* levels upon the knockdown and overexpression of *IRF1* in THP-1 cells (Fig. 5j, k), again supporting the existence of a regulatory loop between *CARINH* and IRF1 as well as their modulation of the downstream anti-inflammatory factor IL-18BP. These findings from human IBD subjects and human cell lines demonstrate that the *CARINH*/IRF1 feedforward loop we observed and characterized in mice is functionally conserved in humans.

### A causal variant in human *CARINH* locus increases IBD risk by impairing the inducible expression of *CARINH*

**Fig. 6:**
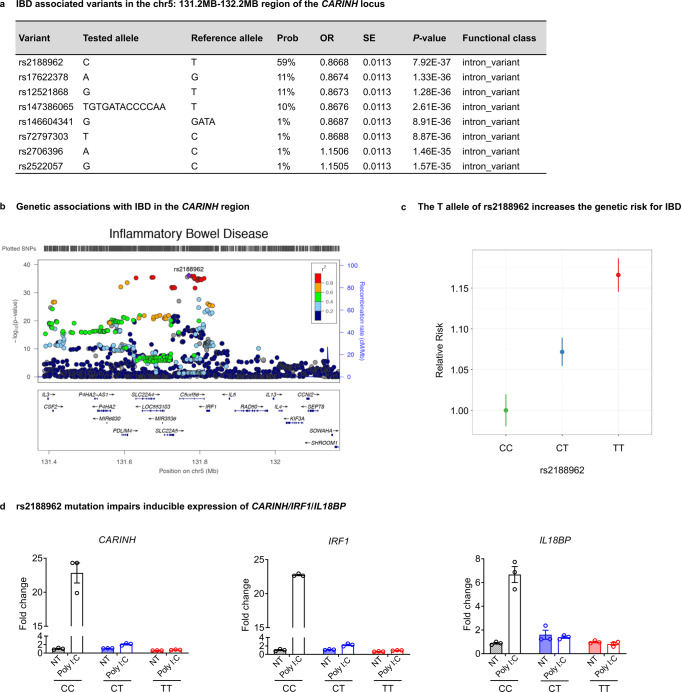
A causal variant in human *CARINH* locus increases IBD risk by impairing the inducible expression of *CARINH*. **a** Summary of IBD-associated variants in the 95% credible set in the chr5: 131.2MB-132.2MB region. Results are taken from reference.^1^ Prob, the posterior probability for a variant to be causal to IBD; OR, odds ratio; SE, standard error. **b** Genetic associations with IBD in the *CARINH* region. *P*-values were taken from an association study of IBD.^1^ The region is refined as 400 kb up- and downstream of the most significantly associated variant: rs2188962. **c** The T allele of rs2188962 increases the genetic risk for IBD. The proportion of individuals diagnosed with IBD for each genotype (CC, CT and TT) of rs2188962 from the International Inflammatory Bowel Disease Genetics Consortium data (35,109 IBD patients and 35,761 controls) were calculated to evaluate the relative risk to IBD. The relative risk was calculated as the ratio of the proportions using genotype CC as the baseline genotype. Error bar indicates 95% confidence interval. **d** qPCR analysis of human *CARINH, IRF1* and *IL18BP* mRNA expression in CRISPR-edited HeLa cell clones with rs2188962 (C/T) variant of each genotype (CC, CT and TT) treated with or without microbe component mimic (Poly I:C). *n* = 3 per group. NT, no treatment. The data in **d** are representative of at least 3 independent experiments. Data represent means ± SEM.

An IBD fine-mapping study has resolved the genetic associations within the Chr5: 131.2MB–132.2MB region to a 95% credible set of eight genetic variants,^2^ all of which reside in the locus of *CARINH* (*C5orf56*; Fig. 6a). On the variant level, this study has pinpointed rs2188962 as the most probable IBD causal variant, with a 59% probability^2^ (Fig. 6a). This variant is located in the intron of *CARINH*, less than 50 kb from the *IRF1* UTR (Fig. 6a, b). The T allele of rs2188962 increases the carrier’s risk of IBD by about 7.5% (Fig. 6c). To further study the functional consequence of the rs2188962 variant, we used the CRISPR technique to mutate C to T of rs2188962 at the *CARINH* locus in a human cell line. Both the C/T and T/T substitutions impaired the Poly I:C (to mimic a microbial signal)-induced expression of *CARINH*, *IRF1*, and *IL18BP* (Fig. 6d). We, therefore, concluded that the T allele of rs2188962 may increase the risk for IBD by affecting the inducible expression of *CARINH* and therefore genes that are regulated by *CARINH*.

## Discussion

IBDs are a set of chronic gastrointestinal inflammatory disorders that affect millions of people worldwide. GWAS works have identified over 200 IBD-associated loci,^57,58^ but few have been conclusively resolved to specific functional variants. While there have been many studies on coding variant determinants of IBD, considerably less attention has been attributed to the study of non-coding variants in IBD (and complex human diseases in general).^59^ In the present study, we found an IBD-associated lncRNA, *CARINH*, and showed that *CARINH* maintains intestinal homeostasis by interacting with the gut microbiota and protecting the host against colitis through the expression of the anti-inflammatory factor IL-18BP.

Although the association between certain lncRNAs and IBD has been reported,^6^ no study has succeeded in providing a mechanistic explanation for how lncRNAs participate in the pathogenesis of IBD. Our results mechanistically highlight the fine regulatory relationship that exists between the lncRNA *Carinh* and its neighboring gene *Irf1* to protect the host against colitis. The specific mechanism is described as follows: *Carinh* RNA promotes *Irf1* expression by interacting with P300/CBP to promote the deposition of activating histone modifications H3K27ac at the *Irf1* locus. The *Carinh*/IRF1 feedforward loop is sustained by microbial factors and functions to maintain microbial homeostasis in the intestine. When colitis is triggered, *Carinh*/IRF1 regulates the induction of the anti-inflammatory factor IL-18BP to prevent further inflammation and colitis exacerbation.

It has been reported that a genomic variant in *CARINH* locus, rs17622517, functions as a condition-specific enhancer for *IRF1*.^60^ In some cases, regulatory elements existing within the non-coding region affect the expression of the neighboring genes.^61^ However, for *CARINH*, there are many different variations which are located in different regions of *CARINH* and associated with different phenotypes and diseases. In our case, rs2188962 in *CARINH* locus has been pinpointed as the most probable IBD causal variant, with a 59% probability (Fig. 6a). We provide experimental evidence that *CARINH* regulates *IRF1* in the form of transcripts. We used CRISPR technique to mutate C to T of rs2188962 at the *CARINH* locus in a human cell line. Both the C/T and T/T substitutions impaired the induced expression of *CARINH*, *IRF1*, and *IL18BP* (Fig. 6d). These results supported that the mutation of rs2188962 affects the transcripts of *CARINH* and genes regulated by *CARINH*.

To exclude the possibility that the defects we observed in *Carinh*^*KO*^ mice were due to the depletion of potential regulatory elements in the *Carinh* locus, we used the siRNA technique to validate the RNA function of *Carinh/CARINH* in both mouse and human cell lines. We found that knocking down *Carinh/CARINH* using specific shRNAs/siRNAs resulted in the decreased expression of *Irf1/IRF1* (Figs. 3l and 5h), indicating that specific RNA transcripts rather than deleted regulatory DNA elements were responsible for the effects observed. Moreover, we also measured the expression of *Irf1* in tissues displaying either high or low expression of *Carinh* in *Carinh*^*KO*^ mice and their WT littermates. We found that the down-regulation of *Irf1* only occurred in tissues associated with high (e.g., BM) but not low *Carinh* expression (e.g., brain) of *Carinh*^*KO*^ mice, in comparison to their WT littermates (Supplementary information, Figs. S8a and S9c). These results suggest that the positive *Carinh/CARINH*-mediated regulation of the neighboring gene *Irf1/IRF1* is due to the regulatory function of *Carinh/CARINH* transcripts and not due to an artifact (i.e., the deletion of the regulatory DNA elements) in *Carinh*^*KO*^ mice. Moreover, by performing ChIP-qPCR experiments using H3K27ac antibody, we found a significant reduction in H3K27ac levels at the *Irf1* promoter of BM cells from *Carinh*^*KO*^ mice compare to that from *Carinh*^*WT*^ mice (Fig. 3n), while there was no difference in H3K4me3 levels at the *Irf1* promoter between *Carinh*^*WT*^ and *Carinh*^*KO*^ cells (Supplementary information, Fig. S10e). These data suggest *Carinh* is involved in the epigenetic regulation of *Irf1* promoter. The RIP experiments in HEK293 cells overexpressing recombinant epigenetic writer proteins and *Carinh* RNA, revealed a strong interaction between the *Carinh* transcript and p300 or CBP, the epigenetic writers of H3K27ac modification (Fig. 3o). Furthemore, the strategy for generating *Carinh*^*KO*^ mice is to delete the exons 3 and 4, two larger exons of *Carinh*. We now have done the RIP experiments analyzing the interaction between CBP/p300 and *Carinh* truncations. We found that full-length *Carinh* RNA but not the RNA transcripts encoded by *Carinh* Exon 1–2 bind to CBP or p300 (Supplementary information, Fig. S11d), indicating the RNA transcript encoded by *Carinh* Exon 3–4 or the complete *Carinh* RNA structure is required for the interaction with these epigenetics modulators. These evidences together suggest that the impaired *Irf1* expression in *Carinh* knockout mice or cells is due to the interrupted binding of *Carinh* RNA to p300 or CBP and the reduced H3K27ac modification at *Irf1* promoter, but is not attributed to the deletion of regulatory DNA elements.

Disease-associated variants at the *CARINH* locus occur at a significantly higher frequency in American (AMR) and European (EUR) populations, than in the East Asian (EAS) population (data from The 1000 Genomes Project). Fine-mapping results have dissected the *CARINH* locus to reveal a credible set of eight variants, among which three have a posterior inclusion probability (PIP) > 10% and are in close linkage disequilibrium (R^2^ > 0.992 from 1000 genomes). Rs2188962, the most probable IBD causal variant found at the *CARINH* locus (59% PIP) has T allele frequencies of 24% and 39% in the AMR and EUR populations, respectively, while at < 1% in the EAS population. Similarly, other two variants with smaller but nontrivial PIPs: rs17622378 (PIP = 11%) and rs12521868 (PIP = 11%), also have significantly higher allele frequencies in the AMR (both 24%) and EUR (both 39%) populations, compared to the EAS population (< 1%). The higher frequency of disease-associated variants at the *CARINH* locus found in the AMR and EUR populations, compared to the EAS population, is consistent with the overall higher incidence of IBD in the AMR and EUR populations.

Like many other lncRNAs, *CARINH* has a tissue-specific expression pattern; it is highly expressed in immune cells and in mucosal tissues (including the intestine and the lung) in both mice and humans (Supplementary information, Fig. S4a and c). Mucosal tissues like the intestine and the lung are readily exposed to pathogenic and commensal microbes and therefore require sophisticated regulatory mechanisms for the modulation of local immune responses and interactions with microbiota. GWAS data have revealed that genetic variations at the *CARINH* locus are highly associated with autoimmune diseases such as IBD and asthma, both of which arise in mucosal tissues that come into contact with diverse microbes.^62,63^ Thus, the continued investigation of the functional and regulatory landscapes of lncRNAs across various mucosal systems is essential to deepen our understanding of the genetic mechanisms underlying the pathogenesis of IBD and other complex/autoimmune disorders, which remain largely unclear despite the many genetic associations discovered to date.

## Materials and methods

### Mice

To generate *Carinh*^*KO*^ mice, exons 3 and 4 of the *Carinh* locus were targeted by two sgRNAs using CRISPR-Cas9 technology (Supplementary information, Fig. S2a). Genotyping of *Carinh*^*KO*^ mice was performed using primers:

5′-CCCTAGCAAGGACAGCTAAG-3′ and 5′-GGTTGAGCAGTCTCTGGATG-3′ for the WT allele; primers: 5′-CCTGTGCCTGTCTCAGTTTG-3′ and 5′-CGGCTAAGGGTTTCAAGTTC-3′ for the targeted allele.

To generate *Irf1*^*KO*^ mice, most of exon 2 and part of exon 3 of the *Irf1* gene were targeted by two sgRNAs using CRISPR-Cas9 technology (Supplementary information, Fig. S8c). Genotyping of *Irf1*^*KO*^ mice was performed using primers:

5′-GGAGAGTGGGGGAGGGTAAT-3′ and 5′-CATAGGTGCATCTCACCCCC-3′ for the targeted allele; primers: 5′-CCTTGGGAGTATGAGCAGGAG-3′ and 5′- CATAGGTGCATCTCACCCCC-3′ for the WT allele.

*Irf1*^*fl/fl*^ mice were generated by inserting two *loxP* sites into either side of the second exon using a Tild-CRISPR (targeted integration with linearized dsDNA-CRISPR)-based strategy as previously described^64^ (Supplementary information, Fig. S8h). Briefly, a PCR-amplified donor which encodes the *loxP* sequence flanked on each side the second exon with 800-bp homology arms is injected with *Cas9* mRNA and sgRNA into mouse zygotes. The pups born were genotyped and the PCR products were sequenced to validate intact integration of the *loxP* sequences into the right genome loci. Genotyping of *Irf1*^*fl/fl*^ mice was performed using primers:

5′-AGGTTCTCAGCACATCCACA-3′ and 5′-CGTCTTGGCTGCCTGTAAC-3′

We crossed *Irf1*^*fl/fl*^ mice with *Villin1-cre*, *Cd4-cre* and *LysM-cre* mice to obtain *Irf1* conditional knockout mice. The *Villin1-cre*, *Cd4-cre* and *LysM-cre* mice were purchased from the Jackson laboratory. The CD45.1 mice and *Rag1*^*−/−*^ mice on a C57BL/6 background were purchased from GemPharmatech Co., Ltd. GF mice were housed in the animal facility at Yale University. cDNA of intestinal tissues was obtained for qPCR detection.

The sex-, age- and background-matched littermates of the knockout or conditional knockout mice were used as the controls in the present study. All mice were on the C57BL/6 background. Mice were maintained in SPF conditions under a strict 12 h light cycle (lights on at 07:00 and off at 19:00). All animal studies were performed according to approved protocols by the Ethics Committee at the University of Science and Technology of China (USTCACUC1801073).

### DSS-induced colitis

To induce colitis, mice were given 2.5% (w/v) DSS (MP Biomedicals) in the drinking water for 6 days, and then switched to regular drinking water until the end of the experiment. Mice were weighed every day to determine percentage body weight changes. Colonoscopy was used to monitor and score the colitis. Colonoscopy was performed in a blinded fashion using the Coloview system (Karl Storz, Germany).^65^ Briefly, colitis score was addressed considering the consistence of stools, granularity of the mucosal surface, translucency of the colon, fibrin deposit and vascularization of the mucosa (0–3 points for each parameter). After sacrifice of the mice, colon length was measured and colon tissue samples were collected for histology. For IL-18BP level analysis in Fig. 3q, harvested colon tissues were homogenated and the IL-18BP levels in colon homogenates were measured using the Mouse interleukin-18 binding protein (IL-18BP) ELISA Kit (CUSABIO, Cat# CSB-E17797m)

### TNBS-induced acute colitis model

Colitis was induced by TNBS as previously described.^66,67^ In brief, 8- to10-week-old male C57BL/6 mice were presensitized 1 week before colitis induction by applying 150 µL presensitization TNBS solution (64% acetone (179124, Sigma-Aldrich), 16% olive oil (Sigma-Aldrich, O1514), 20% 50 mg/mL TNBS (picrylsulfonic acid solution, 5%, Sigma-Aldrich, P2297)) to their preshaved backs. The final concentration of TNBS is 1% (wt/vol). The controls were treated with presensitization solution without TNBS. 1 week after presensitization, presensitized mice were fasted for 12 h, and rectally administered with 100 µL of 2.5% TNBS induction solution (50% ethanol, 50% 50 mg/mL TNBS (picrylsulfonic acid solution, 5%)). The controls were treated only with 50% ethanol. Mouse weight was monitored daily. Mice were sacrificed 72 h after colitis induction. Colon tissues were collected for the measurement of colon length and for histological analysis.

### Generation of radiation BM chimeras

For BM chimera experiments (Fig. 2a), 8- to10-week-old male CD45.1 mice were lethally irradiated (11 Gy) and injected intravenously with 1 × 10^7^ red blood cell-depleted BM cells isolated from either *Carinh*^*KO*^ CD45.2 mice or littermate controls CD45.2 mice 24 h post-irradiation. Recipient mice were given 2 mg/mL neomycin in drinking water for 2 weeks, and BM was allowed to reconstitute in an additional 60 days before administration of DSS. The recipient mice were bled at 60 days post-BM transplant, and the levels of CD45.1 vs CD45.2 cells were measured in order to determine the BM reconstitution efficiency, which was 91%–96%.

For the other BM chimera experiments (Fig. 2e), BM cells from donor mice (CD45.1) were collected and injected intravenously (i.v.) into lethally irradiated recipient mice (*Carinh*^*KO*^ CD45.2 or littermate controls CD45.2 mice). After 60 days, BM reconstitution was verified by staining of peripheral blood cells with PE/Cy7-anti-mouse CD45.1 (clone A20) and FITC-anti-mouse CD45.2 (clone 104) antibodies (Biolegend). The BM reconstitution efficiency was more than 92%. Reconstituted mice were subjected to the DSS-induced colitis described above 60 days after transplantation.

### Administration of clodronate liposomes and anti-CSF-1R antibody

Two days before DSS treatment, 8- to10-week-old male *Carinh*^*KO*^ mice and their littermates were i.p. injected with PBS or 200 µL/20 g body weight of liposomes loaded with clodronate (Liposoma BV, Amsterdam, The Netherlands; Cat#CP-010-010) or a dose of 30 mg/kg (body weight) anti-mouse CSF-1R (CD115) (Clone: AFS98, BioXcell, Cat#BE0213) to deplete phagocytes (clodronate liposomes) and to inhibit the accumulation and infiltration of macrophages.^12,13^

Mice were given 2% (w/v) DSS (MP Biomedicals) in the drinking water at day 0 for 6 days, and then switched to regular drinking water until the end of the experiment (Fig. 2i). To maintain the depletion through the whole DSS treatment period, we injected PBS or clodronate liposomes (clo.) or anti-mouse CSF-1R on day 4 during DSS treatment (Fig. 2i).

### CD45RB^hi^ adoptive transfer colitis

We performed the experiment as described.^68^ Briefly, pure CD4^+^CD25^−^CD45RB^hi^ naive T cells were sorted from 6- to 8-week-old male *Carinh*^*WT*^ and *Carinh*^*KO*^ mice using the BD FACSAria II cell sorter, washed twice with PBS, counted, and i.p. injected 0.5 million cells into each 8- to10-week-old male *Rag1*^*−/−*^ recipient mice. The recipient mice were monitored and weighed each week. H&E staining was performed on paraffin sections of colon previously fixed.

### IL-18BP administration in DSS-induced colitis model

Mouse recombinant IL18BP protein (Sino Biological) was i.p. administered at 0.25 mg/kg twice a day, beginning on the first day of DSS administration and continuing until two days after DSS switching to normal water. This dose of IL-18BP was chosen based on the previous report.^69^

### Histopathology and immunohistochemistry

For histology, colon tissue was fixed in 10% neutral-buffered formalin and embedded in paraffin; 5-µm sections were affixed to slides, deparaffinized and stained with H&E. Morphological changes in the stained sections were examined under a light microscope (BX53, Olympus). H&E-stained sections were blindly scored, the histological scores were determined as described.^70,71^ Briefly, the histological scores include presence of inflammation (0 = none, 1 = slight, 2 = moderate, 3 = severe), extent of inflammation (0 = none, 1 = mucosa, 2 = mucosa + submucosa, 3 = transmural), extent of crypt damage (0 = none, 1 = Basal 1/3 damaged, 2 = Basal 2/3 damaged, 3 = only surface epithelium intact, 4 = entire crypt and epithelium lost), percentage of lesion area (1 = 1%–25%; 2 = 26%–50%; 3 = 51%–75%; 4 = 76%–100%), and the total histopathological score was the summation of the four sections scores.

For immunohistochemistry, colon tissues were fixed with 10% neutral-buffered formalin, embedded in paraffin and cut into 5-μm sections. Sections were deparaffinized with xylene. Microwave heating was used for antigen retrieval. Antibodies against F4/80 (1:300, CST, 70076) and Ki67(1:200, ZSGB-BIO, ZM-0166) were applied and incubated for 1 h at 37 °C. Then HRP-conjugated secondary antibodies were applied and incubated and the positive signals were detected by DAB kit (DAB, Vector, Burlingame, CA). After that, the sections were counterstained with hematoxylin.

### Coding potential verification

To examine coding potential of *CARINH*, the full-length *CARINH* was inserted into the pCirc-GFP-IRES circRNA translation reporter containing a split GFP system using *EcoR*I and *EcoR*V (Supplementary information, Fig. S1d) (provided by Prof. Zefeng Wang, CAS Key Lab for Computational Biology). As previously reported, coding potential of *CARINH* was evaluated by detecting the GFP expression at protein and RNA levels.^72^

Full-length *CARINH* was cloned into pcDNA3.1 with N-terminal start codon ATG and C-terminal HA tag in all three coding patterns and these plasmids were subsequently transfected into HEK293T cells separately (Supplementary information, Fig. S1e). After 48 h, immunoblotting assay was used to detect the HA-tagged protein. ASC with HA tag serves as a positive control.

### RNA-seq

BMDMs from two pairs of 8-week-old male *Carinh*^*WT*^ and *Carinh*^*KO*^ mice were used. Total RNA was isolated with TRIzol reagent (invitrogen, Carlsbad, CA, US). Berrygenomics (Beijing, China) processed the total RNA and constructed the mRNA libraries, and subjected them to standard illumine sequencing on Novaseq 6000 system, and > 40 million Pair-end 150 reads for each sample were obtained. Raw RNA-seq reads were aligned to the mouse genome (mm10, GRCm38) with STAR(v2.5.3a).^73^ Gene expression level and differential analysis was performed with edgeR (v3.29.2).^74^ Genes were considered significantly differentially expressed if showing ≥ 1.5 fold change and FDR < 0.05. Gene set analysis was performed and enriched pathways were obtained through online bioinformatics tools (metascape) and GSEA (v4.0.3).^75^ Volcano plot and pathway plot were gene-rated with R package ‘ggplot2’.

### Cell culture and treatment

HEK293T (ATCC CRL-3216), L929 (ATCC CRL-6364), and J774 (ATCC TIB-67) were obtained from the American Type Culture Collection (ATCC). All of these cells were cultured in Dulbecco’s modified Eagle’s medium (DMEM) (Hyclone) supplemented with 10% fetal bovine serum (FBS) (Clark); THP-1 (ATCC TIB-202) cells were also obtained from ATCC and cultured in RPMI-1640 (Hyclone) medium with 10% FBS (Gibco);

Primary BMDMs were prepared from 8-week-old male mice (C57BL/6 background): *Carinh*^*WT*^ vs *Carinh*^*KO*^ mice and *Irf1*^*WT*^ vs *Irf1*^*KO*^ mice. Briefly, BM has flushed form mouse femurs and cultured in BM media (RPMI-1640 supplemented with 10% heat-inactivated FBS, 30% L929 cell supernatant and Penicillin-Streptomycin) for 7 days to obtain mature, differentiated macrophages. For LPS treatment, BMDMs were plated overnight in 6-well plates at a density of 0.5 million per well and then ultrapure LPS (500 ng/mL) was added at the indicated time points.

All cells were cultured at 37 °C in a 5% CO_2_ incubator.

### Cell transfection and RNA interference

Mouse *Carinh* (NCBI accession #: NR_033332.1) and human *CARINH* (NCBI accession #:NR_161242.1), mouse *Irf1* (NCBI accession #:NM_008390.2 and human *IRF1* (NCBI accession #:NM_002198.3) were cloned into pcDNA3.1 vector. pcDNA3.1-p300-HA and pcDNA3.1-CBP-HA plasmids were provided by Prof. Qiming Sun (Zhejiang University). For transfection experiments, cells were seeded overnight in 6-well plates. The next day, cells were transfected with plasmid DNA using Lipofectamine 2000 as per the manufacturer’s protocol (Invitrogen).

Lipofectamine™ RNAiMAX Transfection Reagent (Invitrogen, Cat#13778075) was used for siRNA knockdown. siRNA and non-targeting control siRNA were purchased from GenePharma, and the experiments were carried out following the manufacturer’s instructions. siRNA targeted sequences are shown in Supplementary information, Table S1.

To generate lentiviruses which express the indicated shRNA for gene knockdown, HEK293T producer cells were transfected with shRNAs (cloned in PLKO.1), pREV, pGag and pVSVG at the ratio of 2:2:2:1 for 48 h. Virus was assembled and released into the supernatant. Lentiviral supernatant was filtered by 0.45-μm filter before infecting target cells. Stably transduced cell lines were selected with puromycin. shRNA primer sequences are shown in Supplementary information, Table S1.

To generate lentiviruses which express mouse *Carinh* or human *CARINH*, HEK293T producer cells were transfected with *Carinh/CARINH* (cloned in pLVX-Puro), pREV, pGag and pVSVG at the ratio of 2:2:2:1 for 48 h. Virus was assembled and released into the supernatant. Lentiviral supernatant was filtered by 0.45 μm filter before infecting target cells. Stably transduced cell lines were selected with puromycin.

### RT-qPCR

For cells and tissues, total RNA was extracted with TRIzol reagent (Invitrogen, Carlsbad, CA, US) in accordance with the manufacturer’s instructions. One microgram of total RNA was reverse transcribed to generate cDNA with Superscript III (Takara, Kusatsu, Shiga, Japan). RT-qPCR was performed using Premix Ex Taq (Takara, Kusatsu, Shiga, Japan) by a Step One Real-Time PCR System (Bio-Rad). The target genes were normalized to the housekeeping gene (*Hprt* or *Gapdh*). Fold changes were presented as a result of 2^-ΔΔCt^. Relative gene expressions were presented as a result of 2^-ΔCt^. Primer sequences were based on Primer-Bank (http://pga.mgh.harvard.edu/primerbank) and blasted to confirm the target genes using Primer-Blast (http://www.ncbi.nlm.nih.gov/tools/primer-blast). The primer sequences used are shown in Supplementary information, Table S1.

### RIP-qPCR

Mouse *Carinh* gene expression plasmid pcDNA3.1-m*Carinh* was co-transfected with pcDNA3.1-p300-HA, or pcDNA3.1-CBP-HA, or empty vector pcDNA3.1-HA in HEK293T cells. Cell lysates were prepared by ultrasonication in RIP Lysis buffer (100 mM KCl, 5 mM MgCl_2_, 10 mM HEPES pH 7.0, 0.5% NP-40, 1 mM dithiothreitol, complete protease inhibitors cocktail and RNase inhibitors).

Cell lysates were immunoprecipitated with anti-HA Magnetic Beads (MedChemExpress) (Supplementary information, Table S3) 6 h at 4 °C with gentle rotation. Beads were pelleted by magnetic field, the supernatant was removed, and beads were resuspended in 500 μL wash buffer (50 mM Tris, pH 7.4, 150 mM NaCl, 1 mM MgCl_2_, 0.05% NP40) and repeated for a total of five times of wash. After elution, co-immunoprecipitated RNA was extracted and analyzed by real-time PCR for the *U1* and m*Carinh*. qPCR primer sequences are listed in Supplementary information, Table S1.

### ChIP assay

BM cells were cross-linked with 1% formaldehyde for 10 min at room temperature while rotating. ChIP assays were then performed by using the ChIP Assay Kit (Beyotime Cat# P2078) with H3K27ac antibody (Abcam Cat#ab4729), H3K4me3 antibody (Abcam Cat#ab8580) and H3K9ac antibody (CST Cat#9649) according to the manufacturer’s instructions. Anti-rabbit immunoglobulin G was used as a negative control. The bound DNA fragments were subjected to RT-qPCR using specific primers (Supplementary information, Table S1).

### 16S rDNA gene sequencing of fecal microbiota

Fecal samples were collected from 6 pairs of male *Carinh*^*WT*^ and *Carinh*^*KO*^ littermates. After 9 weeks, the WT and knockout mice were separated in different cages. Fecal samples from 7 *Irf1*^*WT*^ and 9 *Irf1*^*KO*^ littermates were collected as well. These collected fecal samples were stored at –80 °C until 16 S rRNA gene analysis. DNA was extracted from fecal pellets with a QIAamp stool DNA Mini kit (Qiagen) according to the manufacturer’s instructions. 16S rRNA gene amplicons were generated using the primer pair 515F/806 R as recommended by Earth Microbiome Project.^76^ PCR products were quantified, pooled, cleaned using the PCR Cleanup kit (QIAGEN), and subsequently sequenced on Illumina MiSeq (2 × 250 bp). Custom primers were added to the Illumina MiSeq kit resulting in a 253-bp fragment, and following paired-end joining, sequencing was achieved to a depth of 16,301 ± 14,760 reads (means ± SD). Microbial diversity was analyzed by usearch (v8.1)^77^ and Qiime(v1.9.1).^78^ The linear discriminant analysis effect size Galaxy module (http://huttenhower.sph.harvard.edu/galaxy/) was used for additional statistical analyses.^79^

Read 1: TATGGTAATTGTGTGCCAGCMGCCGCGGTAA

Read 2: AGTCAGTCAGCCGGACTACHVGGGTWTCTAAT

Index sequence primer: ATTAGAWACCCBDGTAGTCCGGCTGACTGACTATTAGAA

### Antibiotic treatments

Mice were treated with ampicillin (1 g/L; BBI), neomycin sulfate (1 g/L; BBI), metronidazole (1 g/L; BBI) and vancomycin (500 mg/L; BBI) in drinking water for 4 weeks. Bacterial removal efficiency was evaluated by quantifying total bacterial DNA in fecal. Then drinking water containing antibiotics was changed to DSS water to induce colitis.

### FMT

FMT was applied as previously described.^80^ In brief, C57BL/6 recipients were treated with ampicillin (1 g/L; BBI), neomycin sulfate (1 g/L; BBI), metronidazole (1 g/L; BBI) and vancomycin (500 mg/L; BBI) in drinking water for 2 weeks and followed with regular water for 2 days. *Carinh*^*KO*^ and *Carinh*^*WT*^ littermate mice were separated when weaned as donors for feces collection. The fresh feces collected from *Carinh*^*KO*^ and *Carinh*^*WT*^ donors were separately resuspended in sterile PBS under anaerobic conditions (Electrotek Scientific Ltd, 85% N_2_, 5% CO_2_, 10% H_2_), vortexed for 3 min and allowed to free settling for 2 min. The supernatant was administered by oral gavage into antibiotic-pretreated C57BL/6 recipients twice a week for 2 weeks. Then the drinking water was changed to 2% DSS water to induce colitis.

### Human samples

All human samples used in the present study were obtained under the approval of the Ethics Committee of the University of Science and Technology of China (USTCEC201900005; Hefei, China). We have obtained informed consent from all participants.

Paraffin sections of colonic resection specimens from IBD patients (*n* = 20, UC = 10, CD = 10) and controls (*n* = 10) were collected from The First Affiliated Hospital of University of Science and Technology of China (Hefei, China). The terminal ileum biopsy specimens from another cohort of 10 controls and 19 CD patients were collected and RNA was extracted for qPCR detection.

Patients were diagnosed on the basis of the standard clinical, endoscopic, and histological criteria of IBD. The colonic resection specimens were obtained during enteroscopy. They were fixed and then made into paraffin sections. The terminal ileum biopsy specimens were collected and RNAs were extracted for qPCR detection. Also, the PBMCs were collected and RNA were extracted for qPCR. The demographic and clinical characteristics of the studied population are shown in Supplementary information, Table S2.

### FISH and immunofluorescent staining

Paraffin sections of colonic resection specimens from IBD patients were used for FISH and immunofluorescent staining.

For FISH, complementary probes targeting human *CARINH* (*C5orf56* (*Homo sapiens* (human)) Gene ID: 441108) were designed, synthesized and labeled by Cy3 (GenePharma). Paraffin sections were first deparaffinized and rehydrated, and then digested with 20 µg/mL proteinase K (Servicebio Cat# G1205) for 20 min. After washing, sections were prehybridized and then hybridized with labeled and mixed RNA probes in hybridization buffer at 37 °C in dark overnight. Nuclei were counterstained with DAPI.

Sequences of RNA probes:

Probe 1: ttttcctccaataggctacaaa

Probe 2: atataccacccagaagtaacca

Probe 3: aggaacatggtttaattgtgca

For immunofluorescent staining, paraffin-embedded sections were first prepared for deparaffinization and rehydration. Heat-induced antigen retrieval using a microwave was followed. After blocking non-specific binding, sections were stained with primary antibodies overnight in a wet chamber at 4 °C in dark. Then sections were washed and stained with secondary antibodies for 50 min at room temperature in dark. Nuclei were counterstained with DAPI. Anti-fade mounting medium were applied onto the slide for the mounting.

Antibodies used in immunofluorescent staining: IRF-1 (D5E4) XP® Rabbit mAb (CST Cat# 8478) (1:200), Anti-CD11b Rabbit pAb (Servicebio Cat# GB11058) (1:500), FITC-conjugated Goat Anti-Rabbit IgG (H + L) (Servicebio Cat# GB22303) (1:200), Cy5-conjugated Goat Anti-Rabbit IgG (H + L) (Servicebio Cat# GB27303) (1:300), Cy3-conjugated Goat Anti-Rabbit IgG (H + L) (Servicebio Cat# GB21303) (1:300).

FISH and Immunofluorescent images were acquired with Pannoramic MIDI (3DHISTECH) and viewed using Caseviewer software (3DHISTECH). Post-acquisition processing (brightness, opacity, contrast, and color balance) was applied to the entire image and accurately reflected the results of the original image.

For FISH statistical analysis in Fig. 5b, 8–10 areas of each single section were randomly selected in 200× magnified images for quantification by ImageJ software (National Institutes of Health, MD, USA) and mean values of each section were shown. Quantification of the FISH results were shown as *CARINH* RNA-positive cell count per mm^2^.

### Generation of rs2188962 mutant human cell lines using CRISPR-Cas9-mediated genome editing

To generate rs2188962 mutant cell lines, the gRNA (5′-GGCCAGTGTTGCCAGAACAC-3′), targeting rs2188962 at *CARINH* locus was cloned into the gRNA-expression plasmid pX330 (Addgene plasmid ID 42230). The donor DNA containing mutation of rs2188962 was synthesized (GenePharma) to replace the genome sequence by homologous recombination. HeLa cells were transfected with pX330-sgRNA plasmid and donor DNA using Lipofectamine 2000 as per the manufacturer’s protocol (Invitrogen). 48 h post-transfection, the GFP^+^ cells (pX330-sgRNA plasmid successfully transfected) were sorted using the BD FACSAria II cell sorter and plated clonally at limiting dilution. The single clones were cultured in 96-well plates for 7 days or longer, depending on the cell growth rate. The genome type of the mutant cells was determined by DNA sequencing.

The donor DNA sequence: 5′-CCATAAACTGCAGCCTGCCTTTTAGCCTTACCTCCTTTGCTCTTGCTCTCTGACCC**t**GTGTTCTGGCAACACTGGCCTGACTACACGCCGTATCACATACAACCAACTGCCCATACCAAC-3′

### Statistical analysis

All values are expressed as means ± SEM. Statistical analysis was performed using unpaired Student’s *t*-tests for two groups, one-way ANOVA and two-way ANOVA (Graphpad Software, San Diego, CA, USA) for multiple groups, with all data points showing a normal distribution. Sample size, number of replicates, and statistical test are indicated in all figure captions. *P* < 0.05 was considered statistically significant.

## Supplementary information

Supplementary information, Fig. S1

Supplementary information, Fig. S2

Supplementary information, Fig. S3

Supplementary information, Fig. S4

Supplementary information, Fig. S5

Supplementary information, Fig. S6

Supplementary information, Fig. S7

Supplementary information, Fig. S8

Supplementary information, Fig. S9

Supplementary information, Fig. S10

Supplementary information, Fig. S11

Supplementary information, Fig. S12

Supplementary information, Fig. S13

Supplementary information, Fig. S14

Supplementary information, Fig. S15

Supplementary information, Table S1

Supplementary information, Table S2

Supplementary information, Table S3

## Data Availability

All the data required for the understanding and critical evaluation of this study are provided in the manuscript and supplementary materials. RNA-seq and 16S rDNA sequencing data are available from the SRA database using accession numbers PRJNA645758 and PRJNA645764, respectively.
