# Supplementary material for: A lncRNA from an inflammatory bowel disease risk locus maintains intestinal host-commensal homeostasis
Source: Cell Res. 2023 Apr 13;33(5):372–88. doi: 10.1038/s41422-023-00790-7 (PMC10156687; doi:10.1038/s41422-023-00790-7)
Supplement: Supplementary file 1 — Supplementary information, Fig. S1 [file 41422_2023_790_MOESM1_ESM.pdf]

Sequence conservation of *Gm12216* (*mCarinh*) and *C5orf56* (*hCARINH*).

a ***Carinh/CARINH* gene location**

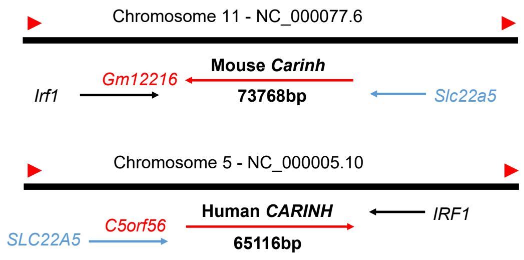

b **TransMap alignment 74.2% identity**

TransMap Ensembl and GENCODE Mappings Version 5 (hg38:ENST00000612967.2-1.1)

TransMap Alignment

Organism	Mouse (Mus musculus)
Genome	mm10
Position	chr11:53771940-53859220
Identity	74.2%
Aligned	88.4%
Chain subset	syn

Source Alignment

Organism	Human (Homo sapiens)
Genome	hg38
Source	Ensembl
Position	chr5:132410919-132488702
Identity	100.0%
Aligned	100.0%
Gene	C5orf56
Gene Id	ENSG00000197536.11
Gene Type	lncRNA
Transcript Id	ENST00000612967.2
Transcript Type	lncRNA
CDS	

Carinh/*CARINH* has no protein coding potential.

c Coding potential prediction by CPAT

Gene ID	Species	Sequence Name	RNA Size	ORF Size	Ficket Score	Hexamer Score	Coding Probability	Coding Label
Gm12216	*Mus musculus*	NR_033332.1	1152	159	0.6214	0.150976463	0.076773846	no
C5orf56	*Homo sapiens*	NR_161242.1	649	354	0.6408	0.003341668	0.093338336	no

d Coding potential verified by circRNA translation reporter system

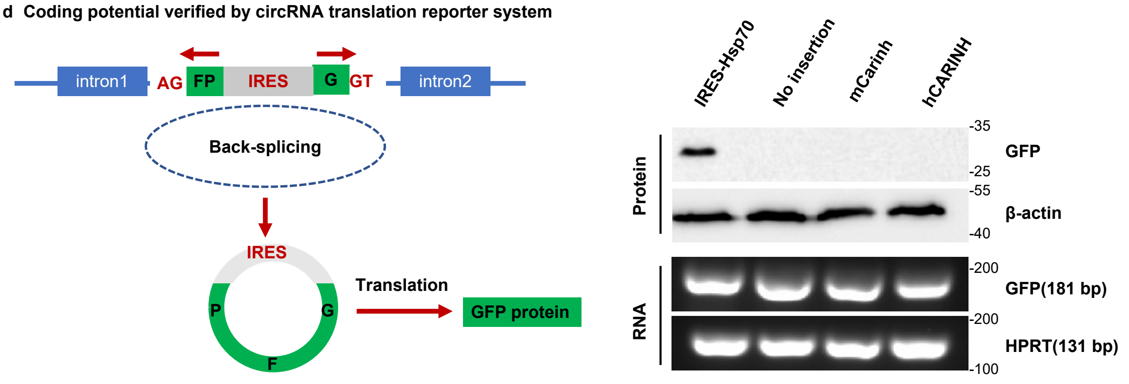

e Coding potential verified by a HA-tagged fusion construct

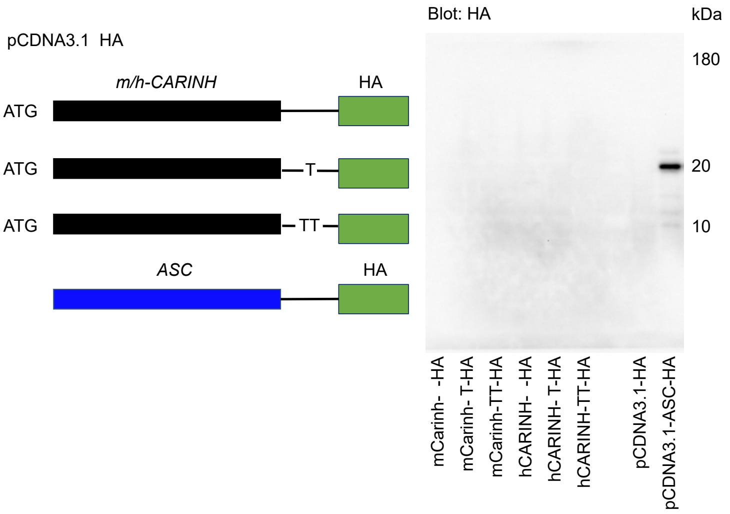

f Ribo-seq analysis for *Irf1* and *Carinh*

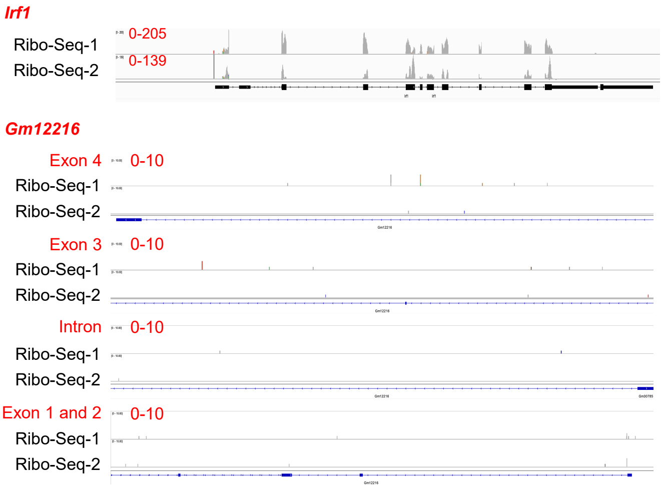

Supplementary information, Fig. S1 Characterization of mouse *Carinh* (*Gm12216*) and human *CARINH* (*C5orf56*).

a-b. Sequence conservation of *Gm12216* (*mCarinh*) and *C5orf56* (*hCARINH*)
a. m*Carinh/hCARINH* genomic location.
b. TransMap Alignment analyzing the identity of mouse *Carinh* and human *CARINH.*

c-f. *Carinh/CARINH* has no protein coding potential
c. Bioinformatics analysis indicated that *Carinh/CARINH* has no coding potential. CPAT prediction of coding potential. http://lilab.research.bcm.edu/cpat/
d. Illustration (left) and application of the *Carinh/CARINH* translation reporter system in HEK293T cells (right) using IRES-Hsp70 and *Carinh/CARINH* plasmids. The mouse *Carinh* and human *CARINH* were cloned into vector containing a split GFP reporter. Translation and transcription of the vector are assayed by western blot and RT-PCR, respectively.
e. Full-length mouse *Carinh* and human *CARINH* were cloned into the eukaryotic expression vector pcDNA3.1 with an N-terminal start codon (ATG) and a C-terminal HA tag in different open reading frames. The coding potential is checked by Western blot of lysates of HEK293T cells transduced with these plasmids. An ASC-HA tag fusion plasmid was used as a positive control.
f. Data analysis of Ribo-seq in *Irf1* and *Carinh* locus. The ribo-seq data are obtained from LPS treated (100 ng/mL for 4 hours) BMDMs in two biological replicates (ribo-seq 1 and ribo-seq 2). The raw data are downloaded from GEO database (Series GSE99787, https://www.ncbi.nlm.nih.gov/geo/query/acc.cgi?acc=GSE99787).

Data (**d** and **e**) are representative of three independent experiments.
