# Supplementary material for: A lncRNA from an inflammatory bowel disease risk locus maintains intestinal host-commensal homeostasis
Source: Cell Res. 2023 Apr 13;33(5):372–88. doi: 10.1038/s41422-023-00790-7 (PMC10156687; doi:10.1038/s41422-023-00790-7)
Supplement: Supplementary file 2 — Supplementary information, Fig. S2 [file 41422_2023_790_MOESM2_ESM.pdf]

Generation and identification of *Carinh* knockout mice.

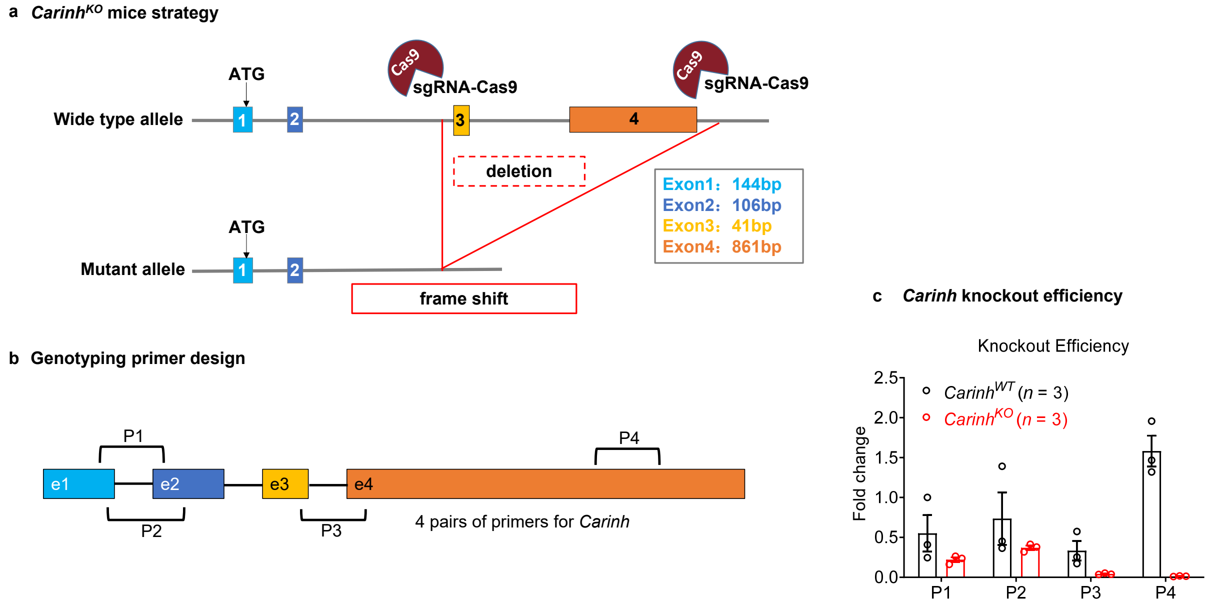

Carinh knockout mice (20 weeks old) show normal body weight and colon histology in steady state.

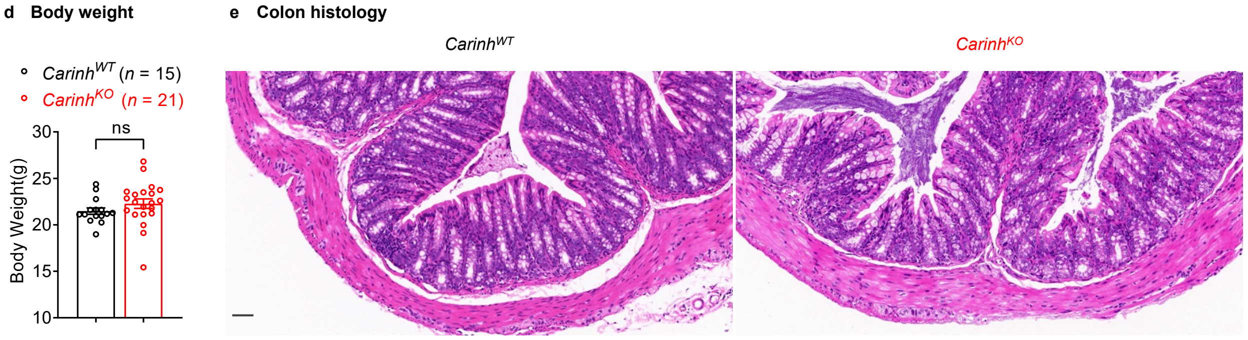

Supplementary information, Fig. S2 Generation and characterization of *Carinh* knockout mice.

a-c. Generation and identification of *Carinh* knockout mice
a. Illustration of the CRISPR–Cas9 strategy to generate *Carinh*^{KO} mice.
b. The designation of 4 pairs of qPCR primers for the validation of *Carinh* RNA expression.
c. qPCR validation of *Carinh* mRNA expression in the *Carinh*^{KO} mice and littermate WT control mice.

d-e. *Carinh* knockout mice (20 weeks old) show normal body weight and colon histology in steady state
There is no difference between body weights (**d**) and colon histology (**e**) of *Carinh*^{WT} and *Carinh*^{KO} mice in steady state.
Data are shown as means ± SEM. Unpaired two-tailed Student's *t*-tests were used for d. ns, not significant. Scale bars, 50μm.
