# Supplementary material for: A lncRNA from an inflammatory bowel disease risk locus maintains intestinal host-commensal homeostasis
Source: Cell Res. 2023 Apr 13;33(5):372–88. doi: 10.1038/s41422-023-00790-7 (PMC10156687; doi:10.1038/s41422-023-00790-7)
Supplement: Supplementary file 3 — Supplementary information, Fig. S3 [file 41422_2023_790_MOESM3_ESM.pdf]

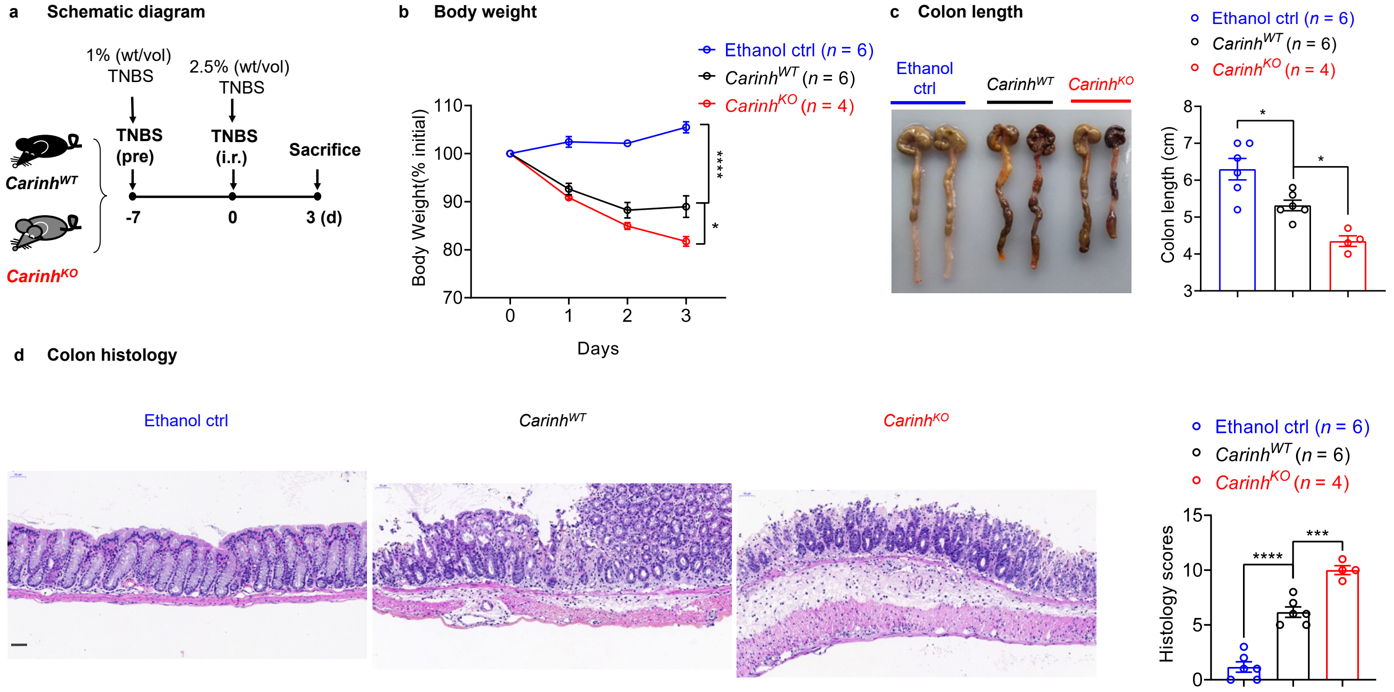

Supplementary information, Fig. S3 ***Carinh*** **protects against TNBS induced colitis.**

a-d. TNBS colitis model was induced in *Carinh*^{WT} and *Carinh*^{KO} mice (**a**). The disease severity was monitored by body weight loss (**b**), colon shortening (**c**), H&E staining of colon tissues (**d**). For H&E staining (**d**): Left, representative pictures. Scale bars, 50μm. Right, quantification of corresponding histology scores. 5 views per mice, Ethanol ctrl n = 6 mice, *Carinh*^{WT} n = 6 mice, *Carinh*^{KO} n = 4 mice.

Data are representative of two independent experiments. Data represent means ± SEM. Body weight changes (**b**) were analyzed by two-way ANOVA. Colon length(**c**) and histology scores (**d**) were analyzed by one-way ANOVA. $^{*}P < 0.05$, $^{***}P < 0.001$, $^{****}P < 0.0001$.
