# Supplementary material for: A lncRNA from an inflammatory bowel disease risk locus maintains intestinal host-commensal homeostasis
Source: Cell Res. 2023 Apr 13;33(5):372–88. doi: 10.1038/s41422-023-00790-7 (PMC10156687; doi:10.1038/s41422-023-00790-7)
Supplement: Supplementary file 4 — Supplementary information, Fig. S4 [file 41422_2023_790_MOESM4_ESM.pdf]

a Expression patterns in mice

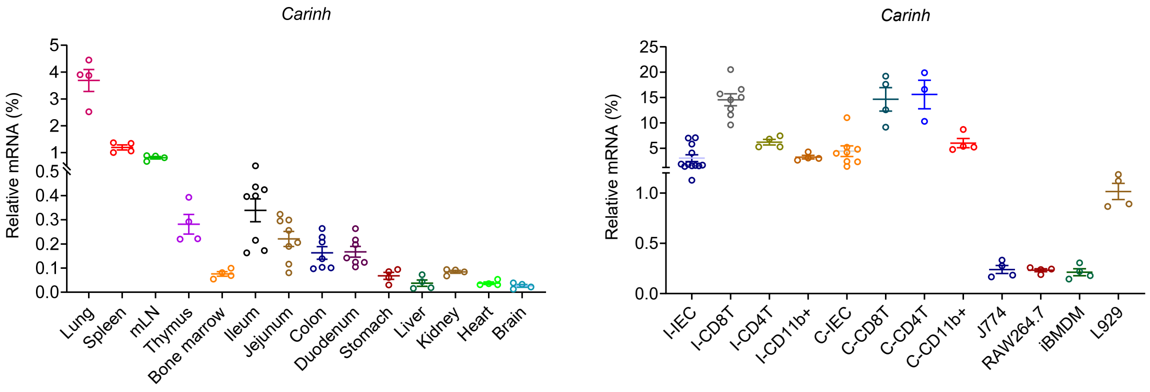

b *Carinh* expression in colonic myeloid cells with or without DSS treatment

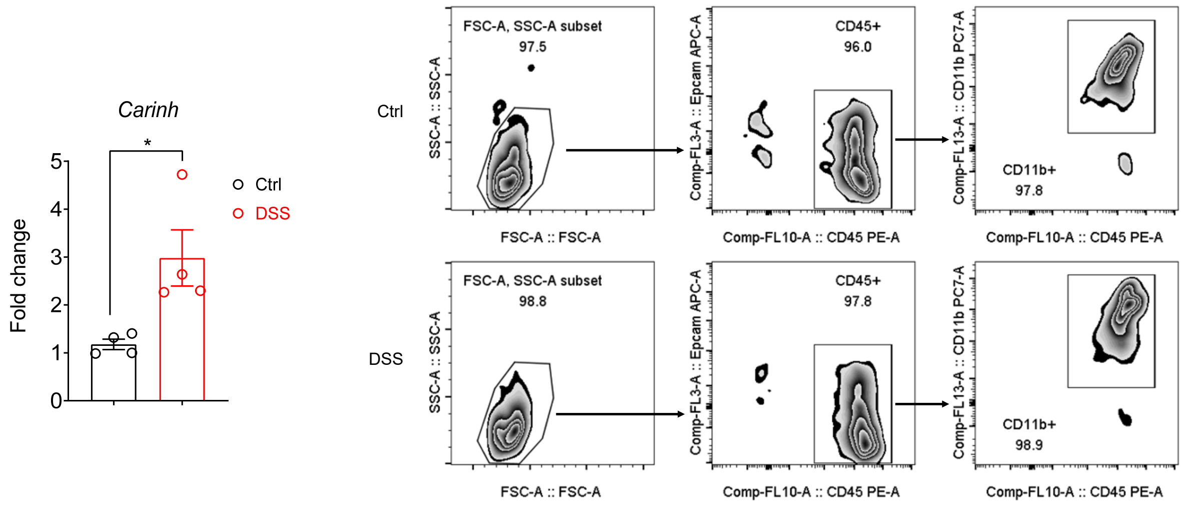

c Expression patterns in human

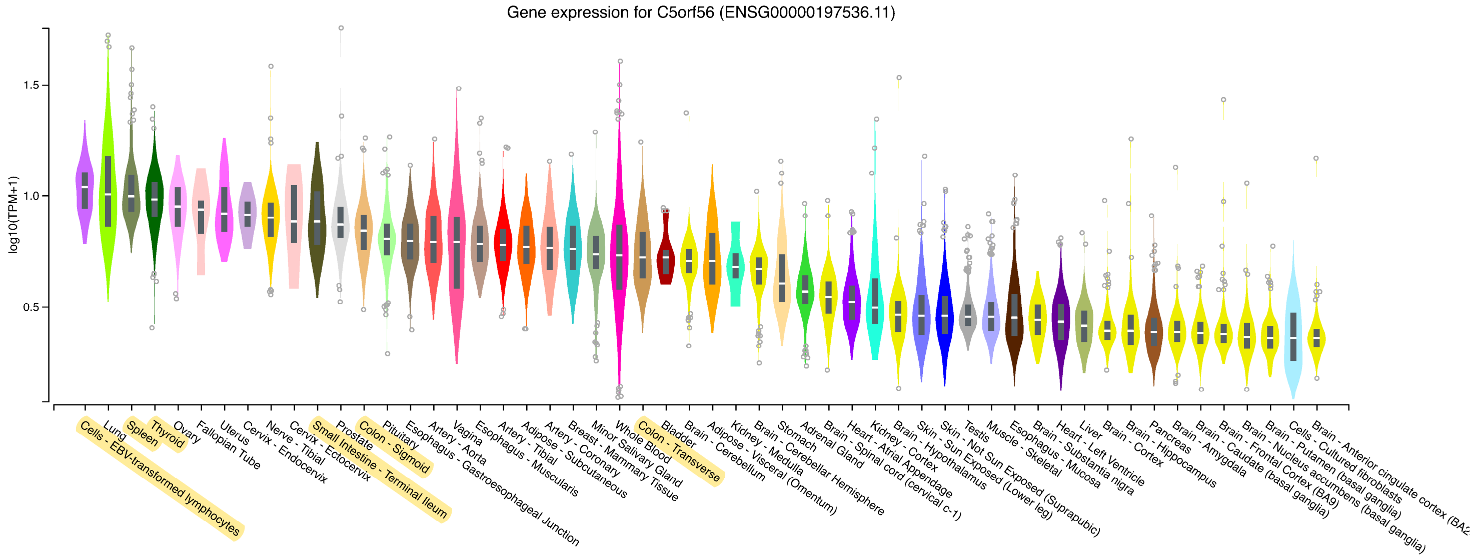

Supplementary information, Fig. S4 *Carinh/CARINH* expression pattern

a. qPCR analysis of *Carinh* expression in the indicated tissues (left panel), primary cells from mice and cell lines (right panel). The data are expressed as percentage of a house keeping gene *Hprt*. Data represent means ± SEM.

b. qPCR analysis of *Carinh* expression in $CD11b^+$ cells sorted from colon of mice without (Ctrl) or with DSS treatment (Left) (n = 4 mice per group). The purity of $CD11b^+$ cells sorted from colon were shown in right panel. Data represented as means ± SEM. Statistical analyses were used unpaired two-tailed Student's t-tests. *P < 0.05. Data are representative of at least 3 independent experiments.

c. Expression profile of *CARINH* across 54 human cell lines and tissues in GTEx. Tissues discussed in the manuscript are highlighted (EBV-transformed lymphocytes, spleen, thyroid, small intestine and colons).
