# Supplementary material for: A lncRNA from an inflammatory bowel disease risk locus maintains intestinal host-commensal homeostasis
Source: Cell Res. 2023 Apr 13;33(5):372–88. doi: 10.1038/s41422-023-00790-7 (PMC10156687; doi:10.1038/s41422-023-00790-7)
Supplement: Supplementary file 5 — Supplementary information, Fig. S5 [file 41422_2023_790_MOESM5_ESM.pdf]

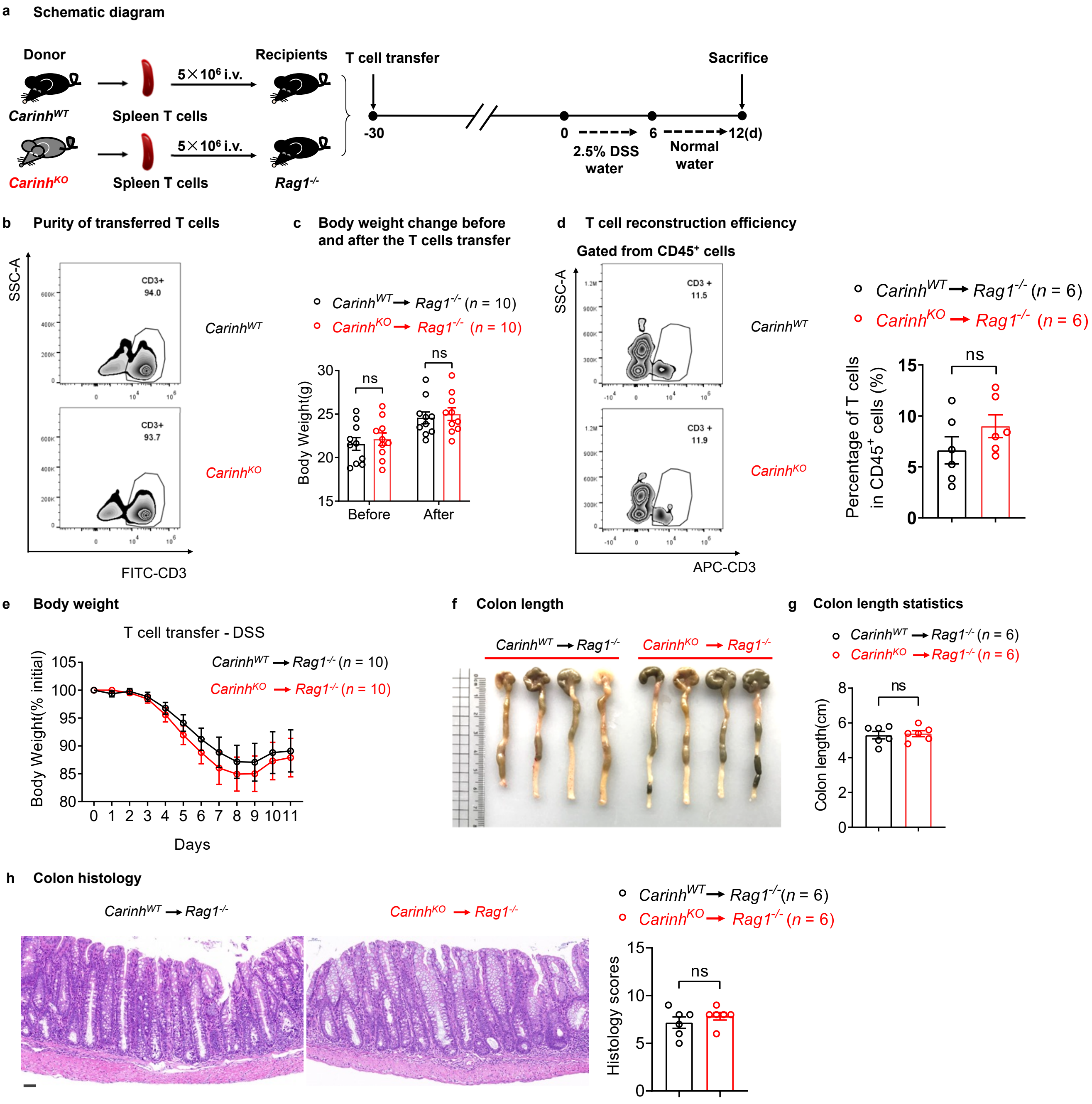

Supplementary information, Fig. S5 ***Carinh*** **deficient T cell-reconstituted mice were not susceptible to DSS-induced colitis**

a-h. *Rag1*$^{-/-}$ mice were adoptively transferred with T cells from *Carinh*^{WT} or *Carinh*^{KO} mice. Four weeks later, the DSS colitis model was induced for these recipient mice (**a**). T cell purity before transfer (**b**) and T cell reconstruction efficiency before DSS treatment (**d**) were analyzed by flow cytometry. Body weight changes before and after T cell transfer were compared between *Carinh*^{WT} and *Carinh*^{KO} T cell transferred recipients (**c**). DSS induced colitis was monitored by body weight loss (**e**), colon shortening (**f**, **g**), and H&E staining of colon tissues (**h**). For H&E staining (**h**): Left, representative pictures. Scale bars, 50μm. Right, quantification of corresponding histology scores. 5 views per mice, 6 mice per group.

Data in (**c**, **e**) are pooled from two independent experiments. Data in (**f**, **h**) and data in (**d**, **g**) are representative of two independent experiments. Data are shown as means ± SEM. Body weight changes (**e**) were analyzed by two-way ANOVA. Unpaired two-tailed Student's *t*-tests were used for other analyses. ns, not significant. Scale bars, 50μm.
