# Supplementary material for: A lncRNA from an inflammatory bowel disease risk locus maintains intestinal host-commensal homeostasis
Source: Cell Res. 2023 Apr 13;33(5):372–88. doi: 10.1038/s41422-023-00790-7 (PMC10156687; doi:10.1038/s41422-023-00790-7)
Supplement: Supplementary file 6 — Supplementary information, Fig. S6 [file 41422_2023_790_MOESM6_ESM.pdf]

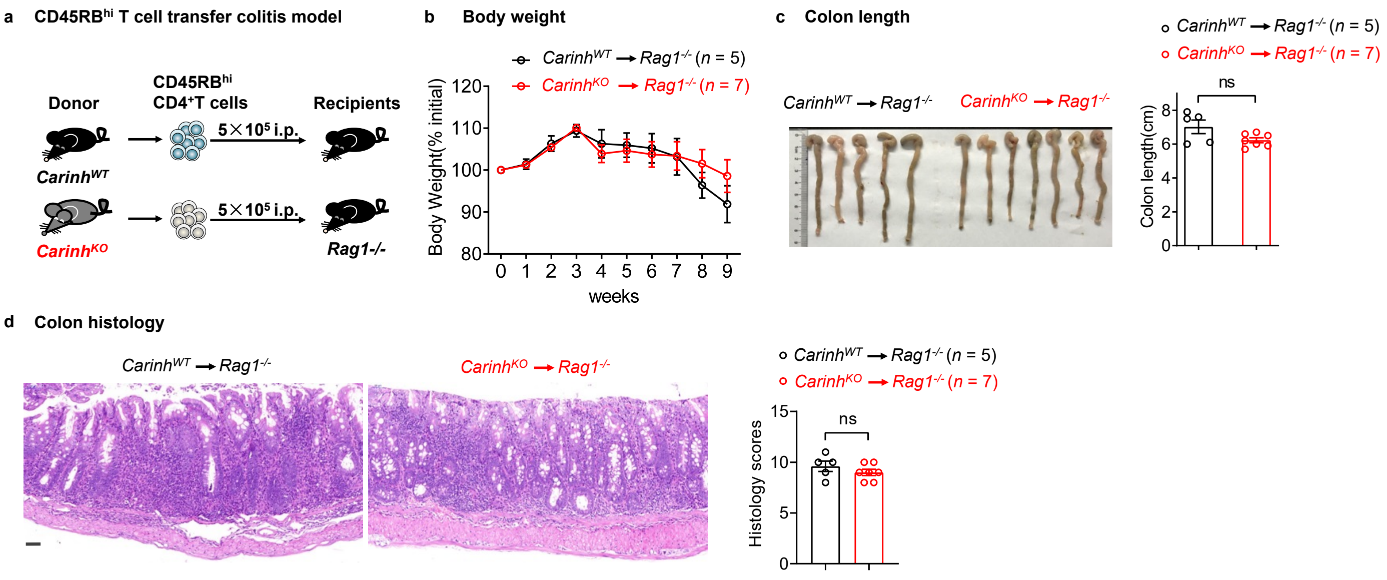

Supplementary information, Fig. S6 ***Carinh*** **deficiency did not affect naïve T cells-transfer induced colitis.**

a-**d**.CD45RB^{hi} naïve CD4^{+}T cells sorted from *Carinh*^{WT} and *Carinh*^{KO} mice, were transferred into *Rag1*$^{-/-}$ mice to induce colitis (**a**). The disease severity was monitored by body weight loss (**b**), colon shortening (**c**), H&E staining of colon tissues (**d**). For H&E staining (d): Left, representative pictures. Scale bars, 50μm. Right, quantification of corresponding histology scores. 5 views per mice, *Carinh*^{WT} to *Rag1*$^{-/-}$ n = 5 mice, *Carinh*^{KO} to *Rag1*$^{-/-}$ n = 7 mice.

Data are representative of two independent experiments. Data are shown as means ± SEM. Body weight changes (**b**) were analyzed by two-way ANOVA. Unpaired two-tailed Student's *t*-tests were used for other analyses. ns, not significant.
