# Supplementary material for: A lncRNA from an inflammatory bowel disease risk locus maintains intestinal host-commensal homeostasis
Source: Cell Res. 2023 Apr 13;33(5):372–88. doi: 10.1038/s41422-023-00790-7 (PMC10156687; doi:10.1038/s41422-023-00790-7)
Supplement: Supplementary file 7 — Supplementary information, Fig. S7 [file 41422_2023_790_MOESM7_ESM.pdf]

a. Myeloid cells depletion assessment in peritoneal cavity

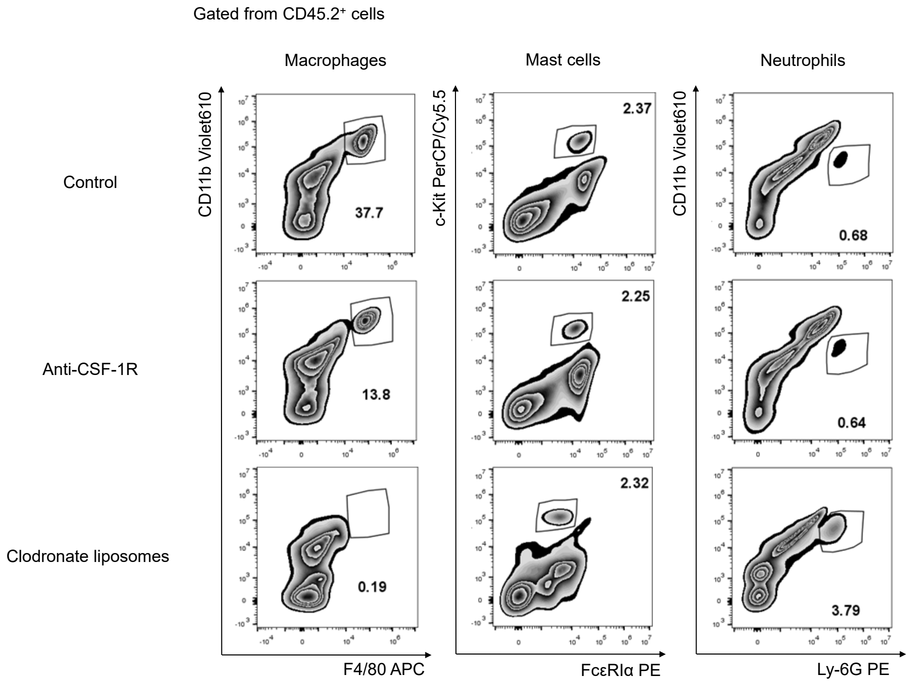

b. Macrophage depletion efficiency assessed in colon (anti-F4/80 immunostaining in colon)

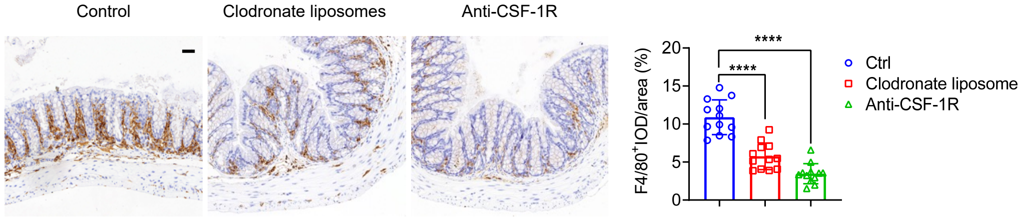

Supplementary information, Fig. S7 Myeloid cells depletion efficiency by Clodronate liposomes or anti-CSF-1R antibody treatment.

a. Depletion efficiency of macrophages, mast cells and neutrophils in peritoneal cavity was assessed by flow cytometry.
b. Depletion efficiency of macrophages was assessed in colon by immunostaining of anti-F4/80 antibody. Left, representative pictures. Scale bars, 50μm. Right, the mean ratios of F4/80$^+$ integrated optical density (IOD) to area (IOD/ area) were used for analyzing the results of immunohistochemistry. n = 12 areas were counted per group. Data represented as means ± SEM. Data in (**b**) was analyzed by one-way ANOVA. ****P < 0.0001.
