# Supplementary material for: A lncRNA from an inflammatory bowel disease risk locus maintains intestinal host-commensal homeostasis
Source: Cell Res. 2023 Apr 13;33(5):372–88. doi: 10.1038/s41422-023-00790-7 (PMC10156687; doi:10.1038/s41422-023-00790-7)
Supplement: Supplementary file 8 — Supplementary information, Fig. S8 [file 41422_2023_790_MOESM8_ESM.pdf]

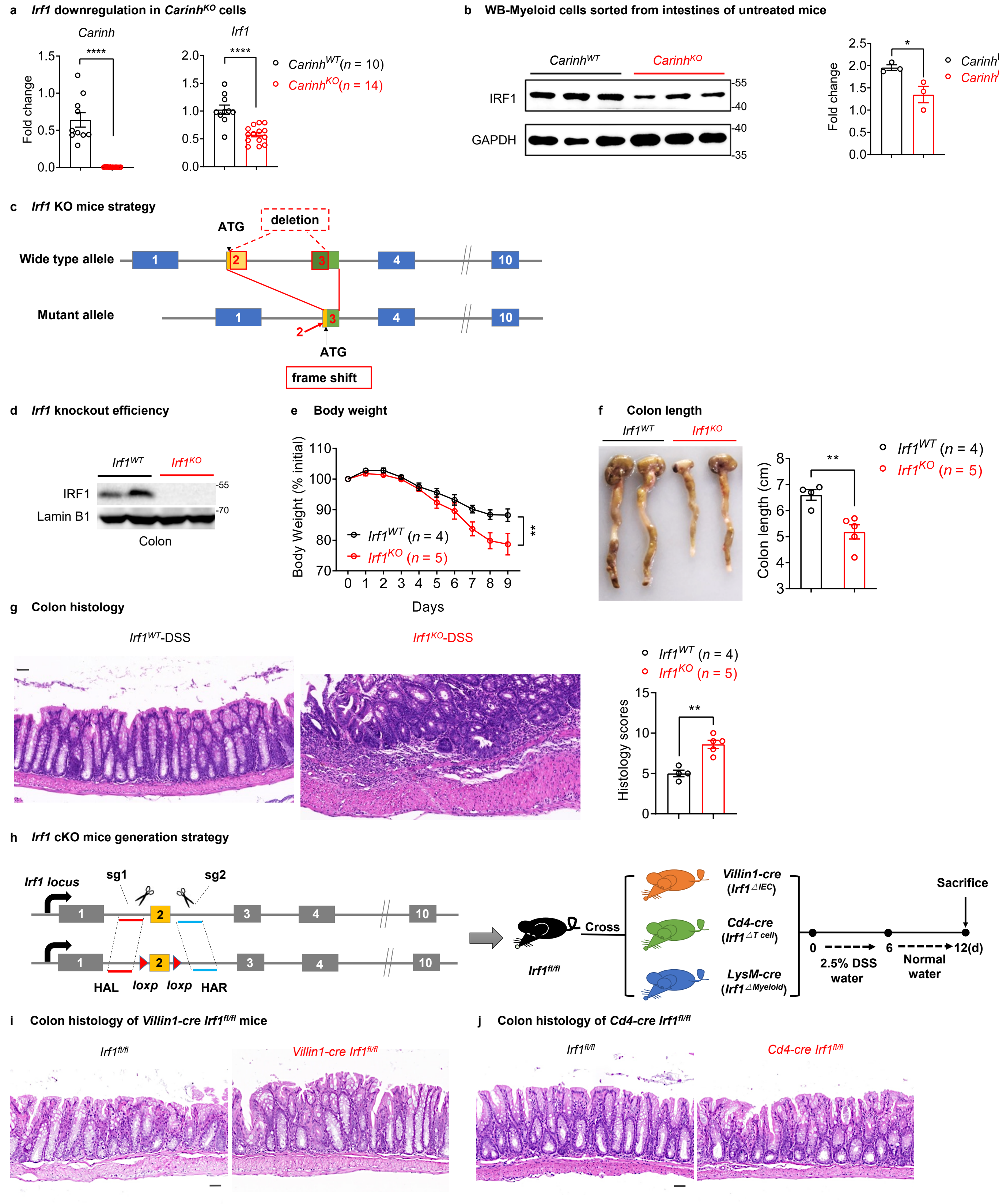

Supplementary information, Fig. S8 IRF1 deficiency in IECs or T cells didn't protect against DSS-induced colitis.

a. Differential *Carinh* and *Irf1* gene expression between *Carinh*^{WT} and *Carinh*^{KO} BM cells was confirmed by qPCR.

b. Western blot detected IRF1 protein expression in CD11b^{+} myeloid cells sorted from intestines of untreated *Carinh*^{WT} and *Carinh*^{KO} mice (n = 3).

c. Knockout strategy for the *Irf1*^{KO} mice

d. *Irf1* knockout efficiency was assessed by western blot analysis of IRF1 protein expression in the colon tissue from *Irf1*^{WT} and *Irf1*^{KO} mice.

e-g. *Irf1*^{WT} and *Irf1*^{KO} mice were given 2.5% DSS for 6 days, colitis was monitored by body weight loss (**e**), colon shortening (**f**) and H&E staining of colon tissues (**g**). For H&E staining (**g**): Left, representative pictures. Scale bars, 50μm. Right, quantification of corresponding histology scores. 5 views per mice, *Irf1*^{WT} n = 4 mice, *Irf1*^{KO} n = 5 mice.

h. Strategy to generate *Irf1-floxed* mice. *Irf1-floxed* mice were crossed with different cell-specific cre lines, including *Villin1-cre* (*Irf1*$^{\triangle IEC}$), *Cd4-cre* (*Irf1*$^{\triangle T\ cell}$), and *LysM-cre* (*Irf1*$^{\triangle Myeloid}$). Then colitis were induced using DSS in these conditional *Irf1* knockout mice.

i-j. Representative pictures of H&E staining of colon tissues in DSS induced *Villin1-cre* (*Irf1*$^{\triangle IEC}$) and *Cd4-cre* (*Irf1*$^{\triangle T\ cell}$) mice were shown in (**i**) and (**j**). Scale bars, 50μm.

Data (**e-g**, **i-j**) are representative of three independent experiments. Data are shown as means ± SEM. Body weight changes (**e**) were analyzed by two-way ANOVA. Unpaired two-tailed Student's *t*-tests were used for other analyses. *P < 0.05, **P < 0.01.
