# Supplementary material for: A lncRNA from an inflammatory bowel disease risk locus maintains intestinal host-commensal homeostasis
Source: Cell Res. 2023 Apr 13;33(5):372–88. doi: 10.1038/s41422-023-00790-7 (PMC10156687; doi:10.1038/s41422-023-00790-7)
Supplement: Supplementary file 9 — Supplementary information, Fig. S9 [file 41422_2023_790_MOESM9_ESM.pdf]

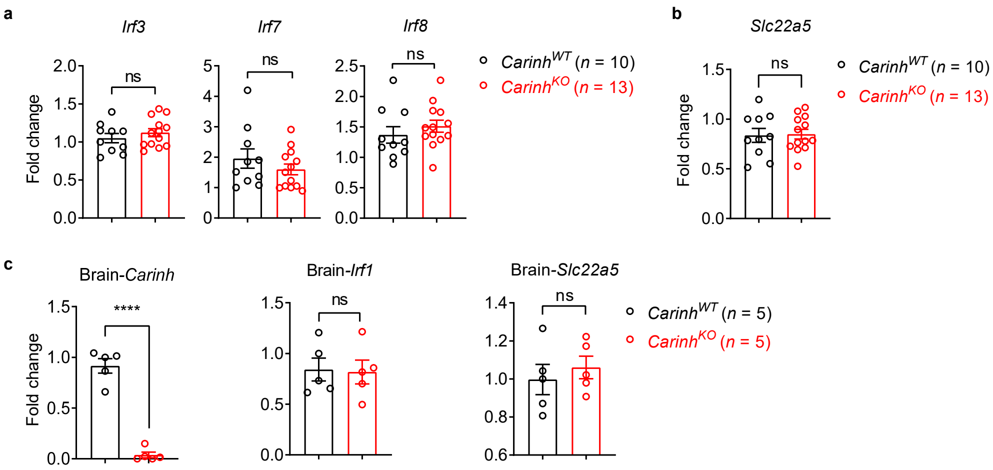

Supplementary information, Fig. S9 qPCR detection of the expression of *Irf* members and neighboring genes of *Carinh*

a-b. qPCR analysis of *Irf3*, *Irf7*, *Irf8* (**a**) and *Slc22a5* (**b**) mRNA expression in BM from *Carinh*^{WT} (n = 10) and *Carinh*^{KO} (n = 13) mice. Data are pooled from 3 independent experiments.

c. qPCR analysis of *Carinh*, *Irf1* and *Slc22a5* mRNA expression in the brain tissues from *Carinh*^{WT} (n = 5) and *Carinh*^{KO} (n = 5) mice. Data are representative of 3 independent experiments.

Data are shown as means ± SEM. Unpaired two-tailed Student's *t*-tests were used for **a-c**. ****P < 0.0001; ns, not significant.
