# Supplementary material for: A lncRNA from an inflammatory bowel disease risk locus maintains intestinal host-commensal homeostasis
Source: Cell Res. 2023 Apr 13;33(5):372–88. doi: 10.1038/s41422-023-00790-7 (PMC10156687; doi:10.1038/s41422-023-00790-7)
Supplement: Supplementary file 10 — Supplementary information, Fig. S10 [file 41422_2023_790_MOESM10_ESM.pdf]

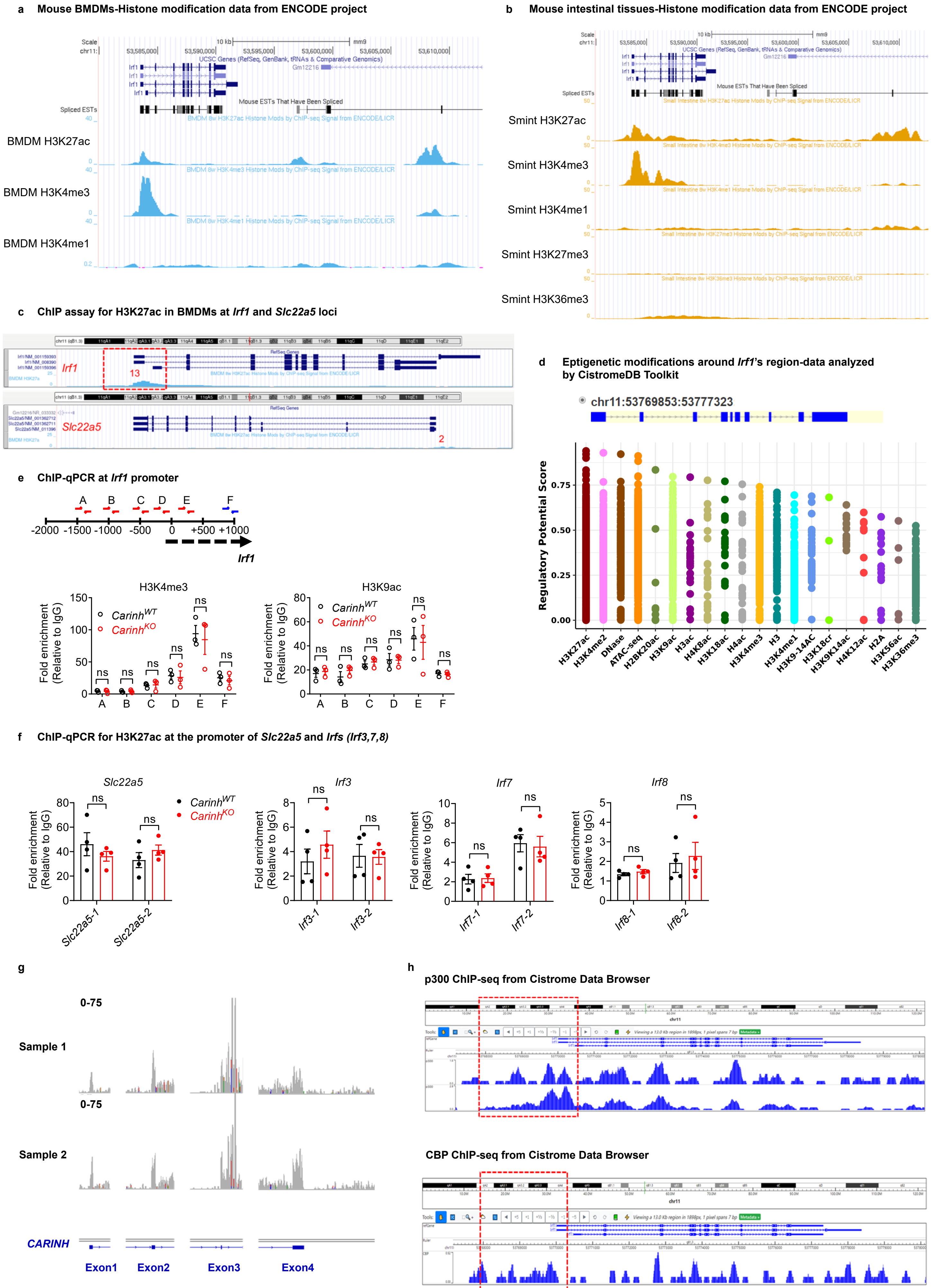

a Mouse BMDMs-Histone modification data from ENCODE project
BMDM H3K27ac
BMDM H3K4me3
BMDM H3K4me1
b Mouse intestinal tissues-Histone modification data from ENCODE project
Smint H3K27ac
Smint H3K4me3
Smint H3K4me1
Smint H3K27me3
Smint H3K36me3
c ChIP assay for H3K27ac in BMDMs at Irf1 and Slc22a5 loci
Irf1
13
Slc22a5
2
d Epigenetic modifications around Irf1's region-data analyzed by CistromeDB Toolkit
chr11:53769853:53777323
Regulatory Potential Score
H3K27ac
H3K4me2
DNase
ATAC-seq
H2BK20ac
H3K9ac
H3ac
H4K8ac
H3K18ac
H4ac
H3K4me3
H3
H3K4me1
H3K9-14AC
H3K18cr
H3K9K14ac
H4K12ac
H2A
H3K56ac
H3K36me3
e ChIP-qPCR at Irf1 promoter
A B C D E F
-2000 -1500 -1000 -500 0 +500 +1000
Irf1
H3K4me3
H3K9ac
Carinh^WT
Carinh^KO
Fold enrichment (Relative to IgG)
ns
f ChIP-qPCR for H3K27ac at the promoter of Slc22a5 and Irfs (Irf3,7,8)
Slc22a5
Irf3
Irf7
Irf8
Slc22a5-1
Slc22a5-2
Irf3-1
Irf3-2
Irf7-1
Irf7-2
Irf8-1
Irf8-2
g
0-75
Sample 1
0-75
Sample 2
CARINH
Exon1
Exon2
Exon3
Exon4
h
p300 ChIP-seq from Cistrome Data Browser
CBP ChIP-seq from Cistrome Data Browser

Supplementary information, Fig. S10 Histone modifications around *Irf1* gene locus

a-b Histone modification data in *Irf1* locus in BMDMs (**a**) and mouse intestinal tissues (**b**) from ENCODE project. Data are shown as reads density mapping around *Irf1* gene loci from UCSC gnome browser.
c. ChIP analysis data from ENCODE project presenting the H3K27ac modification at *Irf1* and *Slc22a5* loci in BMDMs. H3K27ac data are normalized and presented in the same scale.

d. Eptigenetic modifications around *Irf1*'s region. Data analyzed using CistromeDB Toolkit by analyzing public ChIP-seq datasets. http://dbtoolkit.cistrome.org/

e. ChIP–qPCR for H3K4me3 and H3K9ac at the *Irf1* promoter in BM cells. A-E represent selected qPCR primers in the indicated locations at *Irf1* promoter, F represents qPCR primer in non-relevant region. Data are presented as enrichment fold over the IgG control. $n = 3$ per group.

f. ChIP-qPCR for the H3K27ac modification at the promoter of *Slc22a5* and *Irfs (Irf3,7,8)* in BM cells.

g. CBP RIP sequencing data showing the binding of CBP with *Carinh*. Two biological replicates (sample 1 and sample 2) are analyzed from the data of ENCODE (Encyclopedia of DNA Elements) project (https://www.encodeproject.org/experiments/ENCSR974SGV/).

h. Reads density mapping around *Irf1* promoter analyzed from p300 or CBP ChIP-seq through Cistrome Data Brower.
Up panel: Reads density mapping around *Irf1* promoter analyzed from published p300 ChIP-seq data in mouse BMDMs.
Down panel: Reads density mapping around *Irf1* promoter analyzed from published CBP ChIP-seq data in mouse BM hematopoietic progenitor cells.

Data in (**e**) and (**f**) are representative of at least 3 independent experiments. Data represent means ± SEM. Unpaired two-tailed Student's *t*-tests were used for (**e**) and (**f**). ns, not significant.
