# Supplementary material for: A lncRNA from an inflammatory bowel disease risk locus maintains intestinal host-commensal homeostasis
Source: Cell Res. 2023 Apr 13;33(5):372–88. doi: 10.1038/s41422-023-00790-7 (PMC10156687; doi:10.1038/s41422-023-00790-7)
Supplement: Supplementary file 11 — Supplementary information, Fig. S11 [file 41422_2023_790_MOESM11_ESM.pdf]

a ***Gm12216*** **isoforms of CD11b⁺ cells sorted from colon**

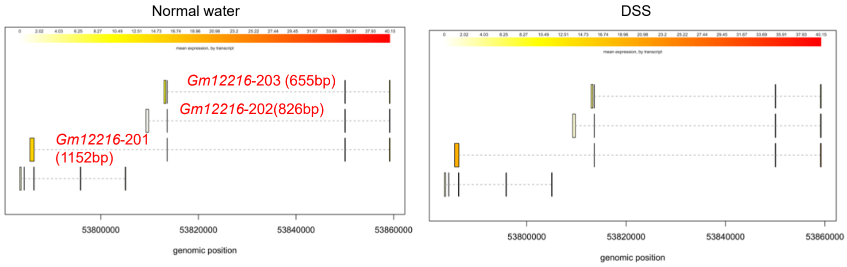

Label	Length (bp)	DSS (cov.)	Normal Water (cov.)
Gm12216-201	1152	19.333157	15.42045867
Gm12216-202	826	1.646165333	0.405168333
Gm12216-203	655	4.711244333	8.573057333
	611	1.158110667	1.640113667

cov: average per-base read coverage

b RIP of ***Gm12216*** **isoforms with CBP or p300**

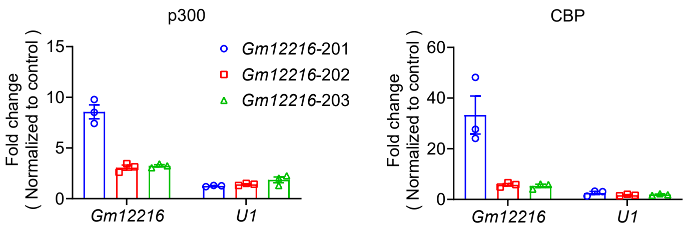

c ***Gm12216*** **isoforms overexpression**

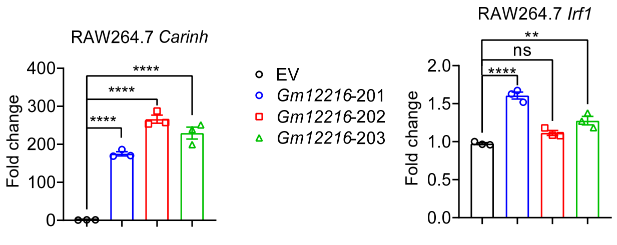

d RIP of Exon 1-2 of ***Carinh*** **with CBP or p300**

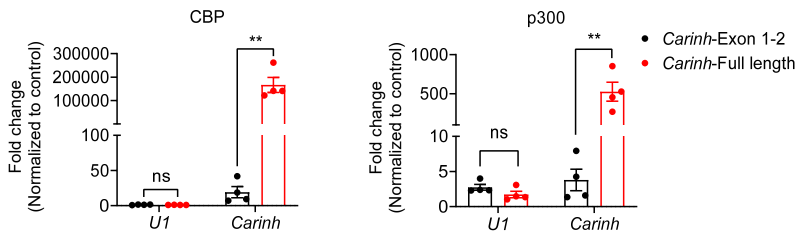

Supplementary information, Fig. S11 Analysis of isoforms of *Gm12216*.

a. Isoforms of *Gm12216* were examined by RNA-seq. CD11b⁺ cells were sorted from colons of mice treated with DSS or water for RNA-seq. Isoforms of *Gm12216* were analyzed and named according to annotations in Ensembl (http://asia.ensembl.org/Mus_musculus/Gene/Summary?db=core;g=ENSMUSG00000081769;r=11:53674244-53750082)

b. RNA-binding protein immunoprecipitation (RIP) analysis between *Gm12216* isoforms and H3K27ac modifier p300/CBP. Protein extracts from HEK293 cells transduced with HA-tagged p300 or HA-tagged CBP and *Gm12216* isoforms, were immunoprecipitated with HA antibodies. *Gm12216* isoforms binds to p300 or CBP were assayed by qPCR. *U1* represents a non-relevant control. Data are presented as relative expression of input and normalized to HA-EV controls. $n = 3$ per group.

c. qPCR analyses of *Carinh* and *Irf1* mRNA expression in RAW264.7 cells transduced with *Gm12216* isoforms plasmid or empty vector. $n = 3$ per group.

d. RIP analysis showed the bindings between the CBP/p300 with full length *Carinh* or truncation. $n = 4$ per group.

Data in **b**, **c**, **d** are representative of at least 3 independent experiments. Data represent means ± SEM.

Data in (**c**) was analyzed by one-way ANOVA. $^{**}P < 0.01$, $^{****}P < 0.0001$, ns, not significant.

Data in (**d**) was analyzed by unpaired two-tailed Student's *t*-tests. $^{**}P < 0.01$, ns, not significant.
