# Supplementary material for: A lncRNA from an inflammatory bowel disease risk locus maintains intestinal host-commensal homeostasis
Source: Cell Res. 2023 Apr 13;33(5):372–88. doi: 10.1038/s41422-023-00790-7 (PMC10156687; doi:10.1038/s41422-023-00790-7)
Supplement: Supplementary file 12 — Supplementary information, Fig. S12 [file 41422_2023_790_MOESM12_ESM.pdf]

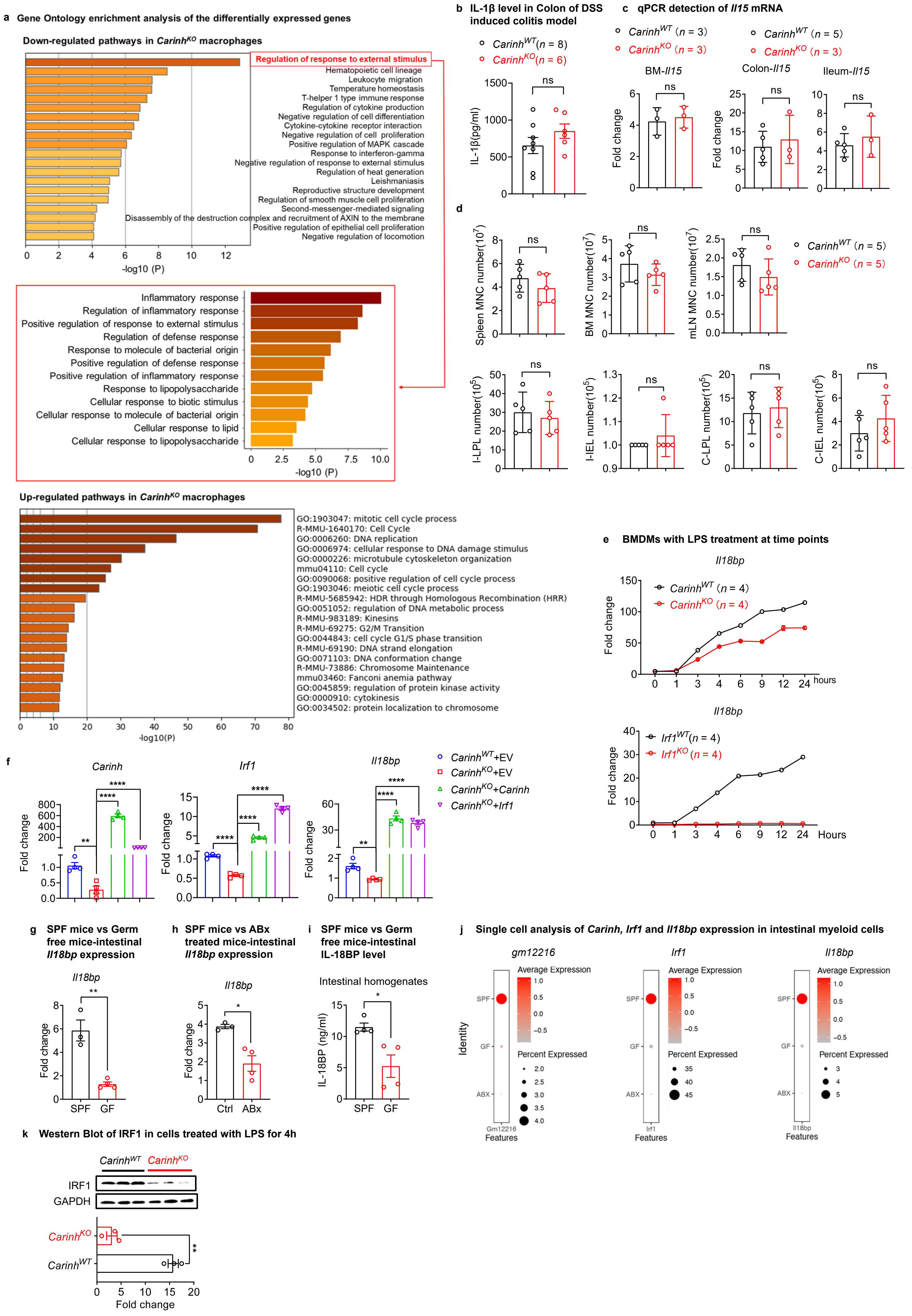
a Gene Ontology enrichment analysis of the differentially expressed genes
Down-regulated pathways in CarinhKO macrophages
Regulation of response to external stimulus
Hematopoietic cell lineage
Leukocyte migration
Temperature homeostasis
T-helper 1 type immune response
Regulation of cytokine production
Negative regulation of cell differentiation
Cytokine-cytokine receptor interaction
Negative regulation of cell proliferation
Positive regulation of MAPK cascade
Response to interferon-gamma
Negative regulation of response to external stimulus
Regulation of heat generation
Leishmaniasis
Reproductive structure development
Regulation of smooth muscle cell proliferation
Second-messenger-mediated signaling
Disassembly of the destruction complex and recruitment of AXIN to the membrane
Positive regulation of epithelial cell proliferation
Negative regulation of locomotion
-log10 (P)
Inflammatory response
Regulation of inflammatory response
Positive regulation of response to external stimulus
Regulation of defense response
Response to molecule of bacterial origin
Positive regulation of defense response
Positive regulation of inflammatory response
Response to lipopolysaccharide
Cellular response to biotic stimulus
Cellular response to molecule of bacterial origin
Cellular response to lipid
Cellular response to lipopolysaccharide
-log10 (P)
Up-regulated pathways in CarinhKO macrophages
GO:1903047: mitotic cell cycle process
R-MMU-1640170: Cell Cycle
GO:0006260: DNA replication
GO:0006974: cellular response to DNA damage stimulus
GO:0000226: microtubule cytoskeleton organization
mmu04110: Cell cycle
GO:0090068: positive regulation of cell cycle process
GO:1903046: meiotic cell cycle process
R-MMU-5685942: HDR through Homologous Recombination (HRR)
GO:0051052: regulation of DNA metabolic process
R-MMU-983189: Kinesins
R-MMU-69275: G2/M Transition
GO:0044843: cell cycle G1/S phase transition
R-MMU-69190: DNA strand elongation
GO:0071103: DNA conformation change
R-MMU-73886: Chromosome Maintenance
mmu03460: Fanconi anemia pathway
GO:0045859: regulation of protein kinase activity
GO:0000910: cytokinesis
GO:0034502: protein localization to chromosome
-log10(P)
b IL-1β level in Colon of DSS induced colitis model
CarinhWT (n = 8)
CarinhKO (n = 6)
IL-1β(pg/ml)
ns
c qPCR detection of Il15 mRNA
CarinhWT (n = 3)
CarinhKO (n = 3)
CarinhWT (n = 5)
CarinhKO (n = 3)
BM-Il15
Colon-Il15
Ileum-Il15
Fold change
d
Spleen MNC number(10^7)
BM MNC number(10^7)
mLN MNC number(10^7)
CarinhWT (n = 5)
CarinhKO (n = 5)
I-LPL number(10^5)
I-IEL number(10^5)
C-LPL number(10^5)
C-IEL number(10^5)
e BMDMs with LPS treatment at time points
Il18bp
CarinhWT (n = 4)
CarinhKO (n = 4)
Fold change
hours
Irf1WT (n = 4)
Irf1KO (n = 4)
Hours
f
Carinh
Irf1
Il18bp
CarinhWT+EV
CarinhKO+EV
CarinhKO+Carinh
CarinhKO+Irf1
Fold change
g SPF mice vs Germ free mice-intestinal Il18bp expression
SPF GF
h SPF mice vs ABx treated mice-intestinal Il18bp expression
Ctrl ABx
i SPF mice vs Germ free mice-intestinal IL-18BP level
Intestinal homogenates
IL-18BP (ng/ml)
SPF GF
j Single cell analysis of Carinh, Irf1 and Il18bp expression in intestinal myeloid cells
gm12216
Irf1
Il18bp
Average Expression
Percent Expressed
Identity
SPF
GF
ABX
Features
k Western Blot of IRF1 in cells treated with LPS for 4h
CarinhWT CarinhKO
IRF1
GAPDH
CarinhKO
CarinhWT
Fold change

Supplementary information, Fig. S12 Intestinal microbiota sustains the expression of *Carinh/Irf1/Il18bp* axis.

a. Gene Ontology enrichment analysis of the differentially expressed genes (down-regulated (up panel) and up-regulated (down panel) in *Carinh*^{KO} BMDMs compared with *Carinh*^{WT} BMDMs; FC > 2.0, FDR <0.05). Up: Summary of down-regulated pathways in *Carinh*^{KO} BMDMs; Middle: members of down-regulated pathways of the first summary above-"Regulation of response to external stimulus".

b. ELISA detection of IL-1β levels in colon homogenates of DSS induced *Carinh*^{WT} (*n* = 8) and *Carinh*^{KO} (*n* = 6) mice.

c. qPCR analysis of *Il15* mRNA expression in the BM cells as well as colon and ileum tissues from *Carinh*^{WT} and *Carinh*^{KO} mice. Data are representative of 3 independent experiments.

d. *Carinh*^{WT} and *Carinh*^{KO} mice were sacrificed and the number of mononuclear cells (MNCs) in spleen, bone marrow (BM), mesenteric lymph nodes (MLNs), as well as the number of ileac lamina propria lymphocytes (I-LPL), ileac intraepithelial lymphocytes (I-IEL), colonic lamina propria lymphocytes (C-LPL) and colonic intraepithelial lymphocytes (C-IEL) were counted and analyzed.

e. qPCR analysis of *Il18bp* mRNA levels in *Carinh*^{KO} BMDMs and *Irf1*^{KO} BMDMs compared with their littermate controls in response to LPS stimulation with time-course. *n* = 4 per group. Data are representative of 3 independent experiments.

f. qPCR analyses of *Carinh*, *Irf1* and *Il18bp* mRNA expression in *Carinh*^{WT} and *Carinh*^{KO} BMDMs transfected with *Carinh* plasmid, *Irf1* plasmid or their corresponding empty vector (EV). *n* = 4 per group. Data are representative of 3 independent experiments.

g. qPCR analysis of *Il18bp* mRNA expression in the intestine of Specific-pathogen-free (SPF) (*n* = 3) and Germ-free (GF) mice (*n* = 4).
h. qPCR analysis of *Il18bp* mRNA expression in the intestine of antibiotic-treated (with ampicillin (1g/L), neomycin sulfate (1g/L), metronidazole (1g/L) and vancomycin (500 mg/L) in drinking water) (*n* = 4) and untreated (*n* = 3) mice.
i. ELISA detection of IL-18BP level in intestinal homogenates of Specific-pathogen-free (SPF) (*n* = 4) and Germ-free (GF) mice (*n* = 4).

j. Analysis from single cell studies of mouse intestines in GEO data base (GSE138902, https://www.ncbi.nlm.nih.gov/geo/query/acc.cgi?acc=GSE138902) . The expression levels of *gm12216, Irf1* and *Il18bp* in CD11b^{+} cells from the colons of SPF mice without ABx, SPF mice with ABx, and GF mice were shown in (**j**).

k. Western blot analysis of IRF1 in *Carinh*^{WT} and *Carinh*^{KO} BMDMs treated with LPS for 4 hours. *n* = 3 per group.

Data (**b-i**, **k**) are representative of at least 3 independent experiments. Data are shown as means ± SEM. Unpaired two-tailed Student's *t*-tests were used for **b-i**, **k**. *$P < 0.05$, **$P < 0.01$, ****$P < 0.0001$, ns, not significant.
