# Supplementary material for: A lncRNA from an inflammatory bowel disease risk locus maintains intestinal host-commensal homeostasis
Source: Cell Res. 2023 Apr 13;33(5):372–88. doi: 10.1038/s41422-023-00790-7 (PMC10156687; doi:10.1038/s41422-023-00790-7)
Supplement: Supplementary file 13 — Supplementary information, Fig. S13 [file 41422_2023_790_MOESM13_ESM.pdf]

a ***Carinh* expression in *Irf1*^{KO} mice**

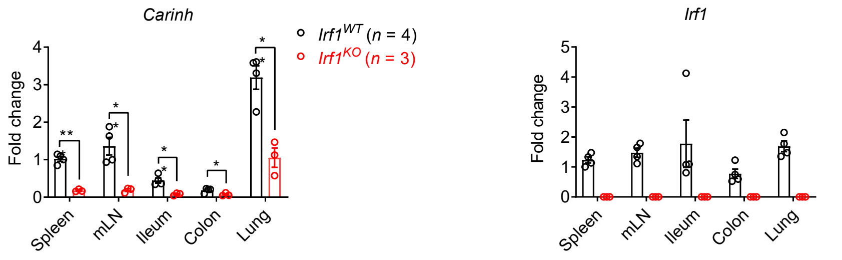

b ***Irf1* overexpression in murine cell lines**

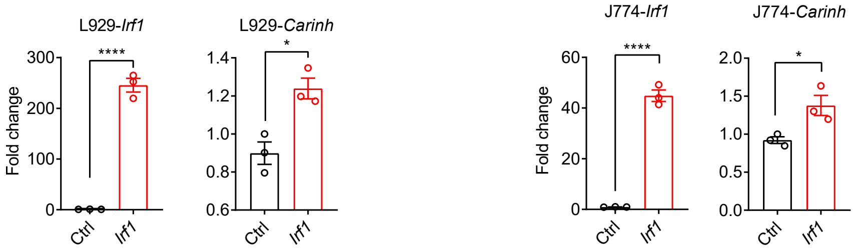

c **IRF1 binding site prediction in mouse *Carinh* promoter**

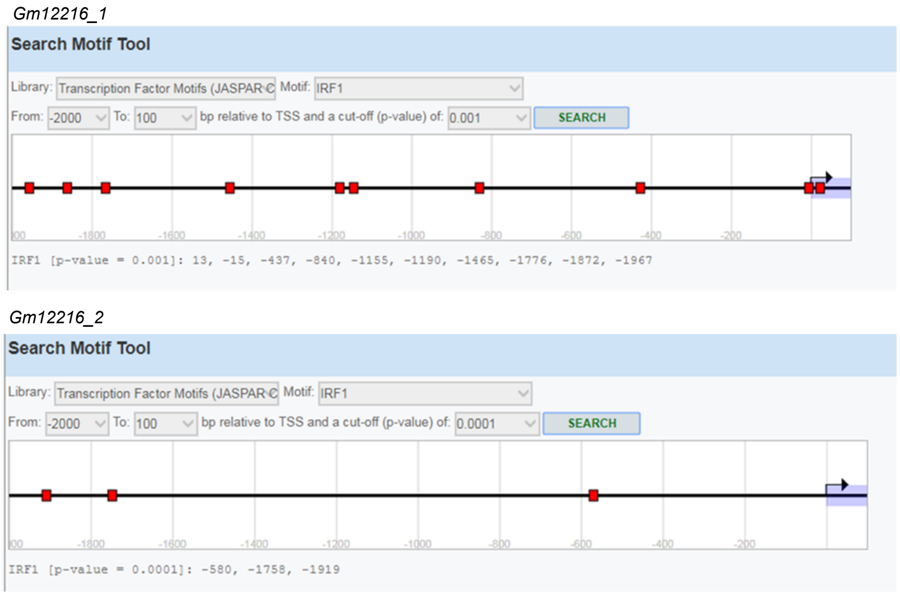

d **IRF1 ChIP-seq from Cistrome Data Browser**

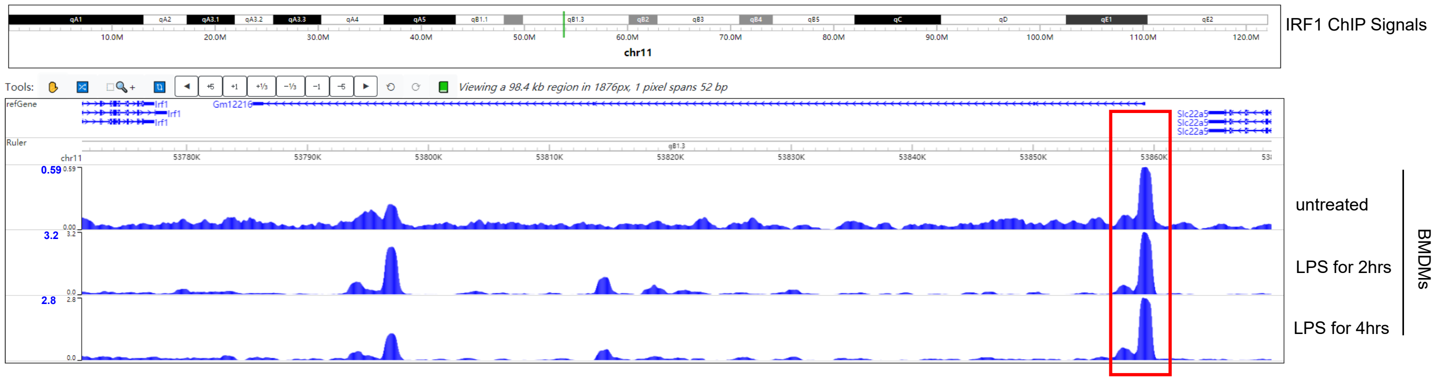

Supplementary information, Fig. S13 IRF1 directly binds to the promoter of the *Carinh* locus and promotes its transcription

a. qPCR analysis of *Carinh* and *Irf1* mRNA expression in the indicated tissues of *Irf1*^{KO} mice and their littermates.
b. qPCR analysis of *Irf1* and *Carinh* mRNA expression in mouse cell lines (L929, J774) with *Irf1* overexpression. *n* = 3 per group.
Data are representative of at least 3 independent experiments. Data are shown as means ± SEM. Unpaired two-tailed Student's *t*-tests were used for **a** and **b**. *$P < 0.05$, **$P < 0.01$, ***$P < 0.001$, ****$P < 0.0001$.

c. Prediction of IRF1 protein binding sites in the promoter of the *Carinh* locus using the online tools: The Eukaryotic Promoter Database. (https://epd.epfl.ch//index.php).
d. Analysis from previously reported ChIP-seq results using an anti-IRF1 antibody in mouse BMDMs with LPS treated 2hrs, 4hrs and untreated controls from the Cistrome Data Browser (http://cistrome.org/db/#/). The analysis of binding sites at *Carinh* promoter region was marked with red line box.
