# Supplementary material for: A lncRNA from an inflammatory bowel disease risk locus maintains intestinal host-commensal homeostasis
Source: Cell Res. 2023 Apr 13;33(5):372–88. doi: 10.1038/s41422-023-00790-7 (PMC10156687; doi:10.1038/s41422-023-00790-7)
Supplement: Supplementary file 14 — Supplementary information, Fig. S14 [file 41422_2023_790_MOESM14_ESM.pdf]

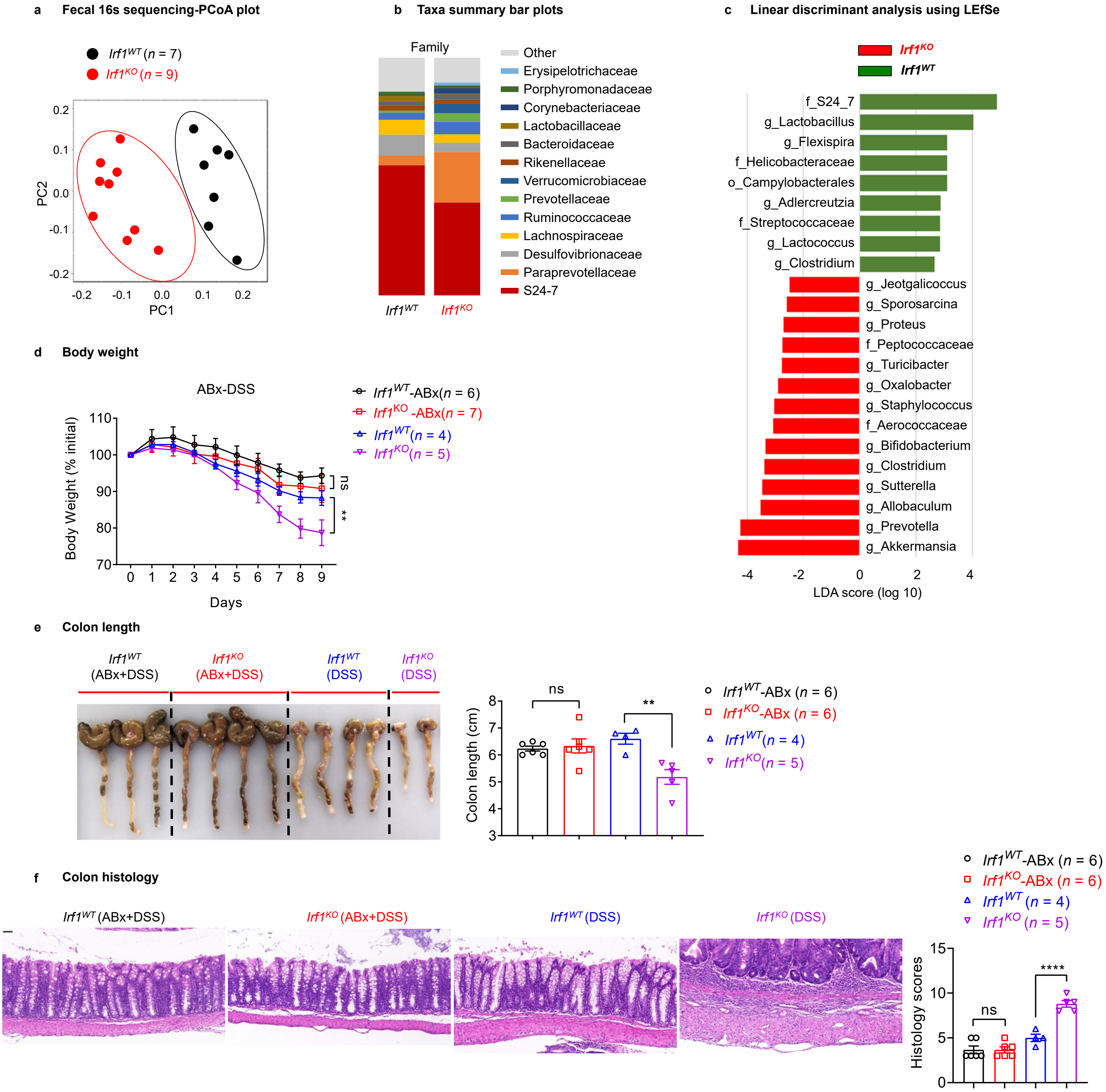

Supplementary information, Fig. S14 Depletion of altered microbiota alleviates the severer colitis in *Irf1*^{KO} mice.

a-c. Fecal 16s rDNA sequencing analysis of *Irf1*^{KO} mice and their littermates.
a. PCoA plot generated from unweighted UniFrac distance matrix displaying the distinct clustering pattern for the intestinal bacteria communities of *Irf1*^{KO} mice (*n* = 9) and of their littermates (*n* = 7).
b. Relative abundance of taxonomic groups averaged across *Irf1*^{KO} mice (*n* = 9) and their littermates (*n* = 7) at the family level.
c. Composition differences of the intestinal microbiota in *Irf1*^{KO} mice and their littermates, determined by linear discriminant analysis using LEfSe.
Irf1^{KO} (*n* = 9) and their littermates *Irf1*^{WT} (*n* = 7) mice were used in 16s rDNA sequencing experiments and were representative of 2 independent 16s rDNA sequencing experiments.

d-f. ABx-treated and untreated *Irf1*^{KO} mice and their littermates were induced with 2.5% DSS. Colitis was monitored by body weight loss (**d**), colon shortening (**e**), and H&E staining of colon tissues (**f**). For H&E staining (f): Left, representative pictures. Scale bars, 50μm. Right, quantification of corresponding histology scores. 5 views per mice.
Data (**d-f**) are representative of 3 independent experiments. Data are shown as means ± SEM. Body weight changes (**d**) was analyzed by two-way ANOVA. Colon length (**e**) and histology scores (**f**) were analyzed by one-way ANOVA. $^{**}P < 0.01$, $^{****}P < 0.0001$, ns, not significant.
