# Supplementary material for: A lncRNA from an inflammatory bowel disease risk locus maintains intestinal host-commensal homeostasis
Source: Cell Res. 2023 Apr 13;33(5):372–88. doi: 10.1038/s41422-023-00790-7 (PMC10156687; doi:10.1038/s41422-023-00790-7)
Supplement: Supplementary file 15 — Supplementary information, Fig. S15 [file 41422_2023_790_MOESM15_ESM.pdf]

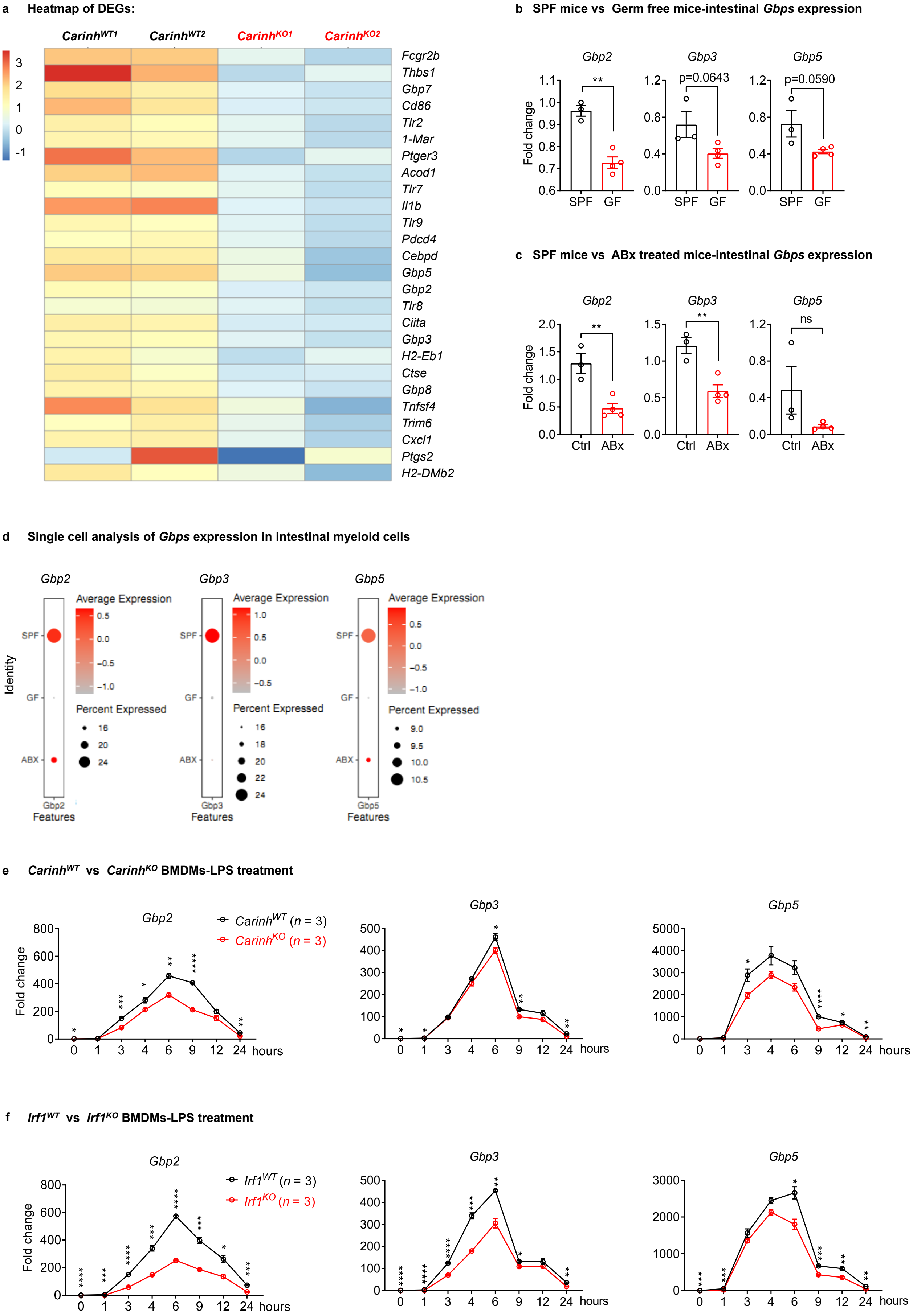
a Heatmap of DEGs:
Carinh^WT1
Carinh^WT2
Carinh^KO1
Carinh^KO2
3
2
1
0
-1
Fcgr2b
Thbs1
Gbp7
Cd86
Tlr2
1-Mar
Ptger3
Acod1
Tlr7
Il1b
Tlr9
Pdcd4
Cebpd
Gbp5
Gbp2
Tlr8
Ciita
Gbp3
H2-Eb1
Ctse
Gbp8
Tnfsf4
Trim6
Cxcl1
Ptgs2
H2-DMb2
b SPF mice vs Germ free mice-intestinal Gbps expression
Gbp2
Gbp3
Gbp5
Fold change
**
p=0.0643
p=0.0590
SPF
GF
c SPF mice vs ABx treated mice-intestinal Gbps expression
Gbp2
Gbp3
Gbp5
Fold change
**
**
ns
Ctrl
ABx
d Single cell analysis of Gbps expression in intestinal myeloid cells
Gbp2
Gbp3
Gbp5
Identity
SPF
GF
ABX
Average Expression
Percent Expressed
Features
e Carinh^WT vs Carinh^KO BMDMs-LPS treatment
Gbp2
Gbp3
Gbp5
Carinh^WT (n = 3)
Carinh^KO (n = 3)
Fold change
hours
f Irf1^WT vs Irf1^KO BMDMs-LPS treatment
Gbp2
Gbp3
Gbp5
Irf1^WT (n = 3)
Irf1^KO (n = 3)
Fold change
hours

Supplementary information, Fig. S15 ***Carinh*** **deficient macrophages showed reduced anti-microbial factors such as GBPs in respond to bacteria products stimulation.**

a. Heatmap showing the down-regulated genes in *Carinh*^{KO} BMDMs compared with *Carinh*^{WT} BMDMs. The differentially expressed genes were identified with a fold change (FC) of *Carinh*^{WT} and *Carinh*^{KO} BMDMs (FC > 2.0, FDR <0.05).

b. qPCR analysis of *Gbp2, Gbp3* and *Gbp5* mRNA expression in the intestine of Specific-pathogen-free (SPF) (n = 3) and Germ-free (GF) mice (n = 4).
c. qPCR analysis of *Gbp2, Gbp3* and *Gbp5* mRNA expression in the intestine of antibiotic-treated (with ampicillin (1g/L), neomycin sulfate (1g/L), metronidazole (1g/L) and vancomycin (500 mg/L) in drinking water) (n = 4) and untreated (n = 3) mice.

d. Analysis from single cell studies of mouse intestines in GEO data base (GSE138902, https://www.ncbi.nlm.nih.gov/geo/query/acc.cgi?acc=GSE138902) . The expression levels of *Gbp2, Gbp3* and *Gbp5* in CD11b^{+} cells from the colons of SPF mice without ABx, SPF mice with ABx, and GF mice were shown in (**d**).

e-f. qPCR analysis of *Gbp2, Gbp3* and *Gbp5* mRNA levels in response to LPS time-course stimulation in *Carinh*^{KO} BMDMs (**e**), *Irf1*^{KO} BMDMs (**f**), and their littermate controls.

Data (**a-c**, **e-f**) are representative of at least 3 independent experiments. Data are shown as means ± SEM. Unpaired two-tailed Student's *t*-tests were used for **b-c**, **e-f**. *P < 0.05, **P < 0.01, ***P < 0.001, ****P < 0.0001; ns, not significant.
