# Supplementary material for: A lncRNA from an inflammatory bowel disease risk locus maintains intestinal host-commensal homeostasis
Source: Cell Res. 2023 Apr 13;33(5):372–88. doi: 10.1038/s41422-023-00790-7 (PMC10156687; doi:10.1038/s41422-023-00790-7)
Supplement: Supplementary file 16 — Supplementary information, Table S1 [file 41422_2023_790_MOESM16_ESM.pdf]

Supplementary information Table S1 List of sequences

1.1 Mouse gene qPCR primer list

Name	Sequence
m-*Carinh* -1F	GACTGTCACGACGGGTGATG
m-*Carinh* -1R	GCTTCCAGGCAAGTCTTCAG
m-*Carinh* -2F	CACGACGGTCGACTGTCAC
m-*Carinh* -2R	CAGCCTCCTGACATCACAAG
m-*Carinh* -3F	AGGAGGCTGGACAGCCAAG
m-*Carinh* -3R	TGGCGGCCTCAGCATCTTC
m-*Carinh* -4F	AGCGCCTCCAACGAGAACAG
m-*Carinh* -4R	CTGGCGGCCTCAGCATCTTC
m-*Carinh* -5F	CCCTAGCAAGGACAGCTAAG
m-*Carinh* -5R	GGTTGAGCAGTCTCTGGATG
m-*Carinh* -6F	CCAGGGAGGGTTCATTTGAG
m-*Carinh* -6R	CGGCAACAGGAAACTGACAC
m-*Irf1* -F	ATGCCAATCACTCGAATGCG
m-*Irf1* -R	CCTGCTTTGTATCGGCCTGT
m-*Gbp2* -F	GGAAAACCCGTCACTTCTGAT
m-*Gbp2* -R	CAGTCGCGGCTCATTAAAGC
m-*Gbp3* -F	TCGGCCCATCACAGAAGATGA
m-*Gbp3* -R	GGAAATTCCGTTCCAGTTGGTT
m-*Gbp4* -F	GAGCAGCTCATCAAAGACCA
m-*Gbp4* -R	TTCCTCACGGAAAGTCTTTTG
m-*Gbp5* -F	CCAGAGTAAAGCGGAACAAG
m-*Gbp5* -R	GTGCAACTCTTGCCTTCTCC
m-*Gbp6* -F	TCATCTTGGTGGTTGGAACA
m-*Gbp6* -R	TCATGAGAAACAATGTCACAAGG
m-*Gbp7* -F	AAACAGCATGAGCACCATCAA
m-*Gbp7* -R	GAAGTGGACTTTGCCCTGAT
m-*Gbp8* -F	TGAGGGTATTTCATCACAGCA
m-*Gbp8* -R	TTGCCAATCTAACTCAGGGATG
m-*Gbp9*- F	ACCGGGAATAGACTGGGTACT
m-*Gbp9* -R	CCGGGCCACACTTGTCATA
m-*Gbp10* -F	CTGTGCAGTCTCAAACCAAG
m-*Gbp10* -R	CACAAGTCGTTCTTAGG
m-*Gbp11* -F	AGCAACTGAGAAGGAAGCTGA
m-*Gbp11* -R	CAAGGAGAGCCTTTTGTTCCT
m-*Slc22a5* -F	ACTGTGCCAGGGGTGCTAT
m-*Slc22a5* -R	TCCGTGTTCGGATCAGATCATAA
m-*Irf3* -F	GAGAGCCGAACGAGGTTCAG
m-*Irf3* -R	CTTCCAGGTTGACACGTCCG
m-*Irf8* -F	CGGGGCTGATCTGGGAAAAT
m-*Irf8* -R	CACAGCGTAACCTCGTCTTC
m-*Hprt* -F	GATTAGCGATGATGAACCAGGTT
m-*Hprt*- R	CCTCCCATCTCCTTCATGACA
m-*Reg3γ* -F	CAGACAAGATGCTTCCCCGT
m-*Reg3γ* -R	GCAACTTCACCTTGCACCTG
m-*Il18bp* -F	GAGGGCCACACAAGTCGC
m-*Il18bp* -R	GCTGGGCCAGAATGATGTGA
Gfp -F	TACGGCGTGCAGTGCTTCAG
Gfp -R	TCAGCTCGATGCGGTTCACC

Supplementary information Table S1 List of sequences

1.2 Human gene qPCR primer list

Name	Sequence
h-*SLC22A5* -F	CACCATTGTGACCGAGCAAGA
h-*SLC22A5* -R	TTCCAGTCGTCCTCACACACC
h-*CARINH* -F3	GGGTGCCAGAGTTTGTAGCC
h-*CARINH* -R3	AATGCTGCAAGCAGGAGTCTA
h-*IL18BP* -F	CAGCTCTGGGCTGGGCTGAG
h-*IL18BP* -R	GGGGTGTGTTGCGCATCCAC
h-*IRF1* -F2	CTGTGCGAGTGTACCGGATG
h-*IRF1* -R2	ATCCCCACATGACTTCCTCTT

Supplementary information Table S1 List of sequences

1.3 ChIP-qPCR primer list

Name	Sequence
U1 -F	ATACTTACCTGGCAGGGGAG
U1 -R	CAGGGGAAAGCGCGAACGCA
Irf1 -H3K27ac-A-F	ACATTCAAACTGGGTGCTGT
Irf1 -H3K27ac-A-R	CTCAGGCTTCTTCATTCGCT
Irf1 -H3K27ac-B-F	AGGAATAAGGGTCGCCTTG
Irf1 -H3K27ac-B-R	ACTTGCTAGTCTGAAGTGATG
Irf1 -H3K27ac-C-F	CGTGGAGAAGGGGAAGACCA
Irf1 -H3K27ac-C-R	CCTTGCTCCACTCCACTC
Irf1 -H3K27ac-D-F	CGAAATGATGAGGCCGAGT
Irf1 -H3K27ac-D-R	CTAAACACTTAGCGGGATTCC
Irf1 -H3K27ac-E-F	ACGTGCTTTCACAGTCTAAGCC
Irf1 -H3K27ac-E-R	CAGGAGCGATTCGCAGAGG
Irf1 -H3K27ac-F-F	GACTGAAAAGAATGGGCAGGTGG
Irf1 -H3K27ac-F-R	CCTCACACCCAGGTCATTACC
Slc22a5 -H3K27ac-F1	GCACTGCCAGGGAGGAAGAACAC
Slc22a5 -H3K27ac-R1	AAGGCCATGGAACAGATGCTGCC
Slc22a5 -H3K27ac-F2	CTCCGCAGCCACAAAAGATGGC
Slc22a5 -H3K27ac-R2	AGGGGGCGGAATTGGGTCTTTTC
Irf3 -H3K27ac-F1	GCTTGTGCTGAAGCTGAACCGT
Irf3 -H3K27ac-R1	GAAGGGATCACTTGCCCAGAC
Irf3 -H3K27ac-F2	AGGGCGCCTAACTCCAAGGA
Irf3 -H3K27ac-R2	GAAATCGGACCAGCGTTTGCC
Irf7 -H3K27ac-F1	TGGGTGCTGGATTTGAACCCCTG
Irf7 -H3K27ac-R1	CTGGCTGTCCTGGAACTCACTC
Irf7 -H3K27ac-F2	GAACAGGAGGGAAGGATTTGCA
Irf7 -H3K27ac-R2	GACAAGAATTTCCCCTGTGGCA
Irf8 -H3K27ac-F1	TCTAGAACACTTCGGGGCCT
Irf8 -H3K27ac-R1	TGTACATTTCCCACACGCCA
Irf8 -H3K27ac-F2	CGCCCCCGGAGTAAAGAGAGA
Irf8 -H3K27ac-R2	ACCCCATCGCGTCCTACCTG

Supplementary information Table S1 List of sequences

1.4 shRNA sequence list

**Name**	**Sequence**
m*Carinh* -F1	CCGGGCTATCCTCAGGGTGTATAGCGGATCCGCTATACACCCTGAGGATAGCtttttg
m*Carinh* -R1	aattcaaaaaGCTATCCTCAGGGTGTATAGCGGATCCGCTATACACCCTGAGGATAGC
m*Carinh* -F2	CCGGGCTCTAGGAAACAGCCTATGGGGATCCCCATAGGCTGTTTCCTAGAGCtttttg
m*Carinh* -R2	aattcaaaaaGCTCTAGGAAACAGCCTATGGGGATCCCCATAGGCTGTTTCCTAGAGC
m*Carinh* -F3	CCGGGGAGTGTCAGTTTCCTGTTGCGGATCCGCAACAGGAAACTGACACTCCtttttg
m*Carinh* -R3	aattcaaaaaGGAGTGTCAGTTTCCTGTTGCGGATCCGCAACAGGAAACTGACACTCC
h*CARINH* -F1	CCGGGCCAGTACCAGGAAAGGATAGGGATCCCTATCCTTTCCTGGTACTGGCtttttg
h*CARINH* -R1	aattcaaaaaGCCAGTACCAGGAAAGGATAGGGATCCCTATCCTTTCCTGGTACTGGC
h*CARINH* -F2	CCGGGCAGAGCTCCCAGATATATGCGGATCCGCATATATCTGGGAGCTCTGCtttttg
h*CARINH* -R2	aattcaaaaaGCAGAGCTCCCAGATATATGCGGATCCGCATATATCTGGGAGCTCTGC
h*CARINH* -F3	CCGGGGTATATCGTAGGTGGCTTTAGGATCCTAAAGCCACCTACGATATACCtttttg
h*CARINH* -R3	aattcaaaaaGGTATATCGTAGGTGGCTTTAGGATCCTAAAGCCACCTACGATATACC

Supplementary information Table S1 List of sequences

1.5 siRNA sequence list

Name	Sense	Anti-sense
IRF1 -homo-617	GCCGAGAUGCUAAGAGCAATT	UUGCUCUUAGCAUCUCGGCTT
IRF1 -homo-722	GCUACACAGUUCCAGGCUATT	UAGCCUGGAACUGUGUAGCTT
IRF1 -homo-1031	CCUCUGUCUAUGGAGACUUTT	AAGUCUCCAUAGACAGAGGTT
Gm12216 -mus-309	CCAACAUCGCUUCCACUUUTT	AAAGUGGAAGCGAUGUUGGTT
Gm12216 -mus-662	GCCUAUCCCAGGCUUUGAUTT	AUCAAAGCCUGGGAUAGGCTT
Gm12216 -mus-996	GCUAUCCUCAGGGUGUAUATT	GCUAUCCUCAGGGUGUAUATT
C5orf56 -homo-507	GCCAGUACCAGGAAAGGAUTT	AUCCUUUCCUGGUACUGGCTT
C5orf56 -homo-598	GCUCCCAGAUAUAUGCACATT	UGUGCAUAUAUCUGGGAGCTT
C5orf56 -homo-355	GCUGGAGAGUUGAAUGCCATT	UGGCAUUCAACUCUCCAGCTT
