# Supplementary material for: A lncRNA from an inflammatory bowel disease risk locus maintains intestinal host-commensal homeostasis
Source: Cell Res. 2023 Apr 13;33(5):372–88. doi: 10.1038/s41422-023-00790-7 (PMC10156687; doi:10.1038/s41422-023-00790-7)
Supplement: Supplementary file 17 — Supplementary information, Table S2 [file 41422_2023_790_MOESM17_ESM.pdf]

Supplementary information Table S2 Demographic and clinical characteristics of the studied population

**Pathological diagnosis**	**CD**	**UC**	**Controls**
Num.	29	10	21
Male, n (%)	17 (59)	5 (50)	12(57)
Age, yr	29.9±14.4 (14-68)	47.9±15.3 (17-66)	46.4±18.4(16-83)
Disease duration, mo	14.1±12.7 (1-72)	53.4±58.1 (2-240)	-
**Disease phenotype, n (%)**			
B1	8 (28)	-	-
B2	5 (17)	-	-
B3	1 (3)	-	-
B1p	11 (38)	-	-
B2p	2 (7)	-	-
B3p	2 (7)	-	-
**Disease location, n (%)**			
L1	9 (31)	-	-
L2	1 (3)	-	-
L3	19 (66)	-	-
E2	-	3 (30)	-
E3	-	7 (70)	-
**Medication, n (%)**			
Mesalazine	3 (10.5)	3 (30)	-
Adalimumab	1 (3)	-	-
Enteral nutrition	1 (3)	-	-
Infliximab	11 (38)	-	-
Prednisone	3 (10.5)	1 (10)	-
Sulfasalazine (SASP)	-	1 (10)	-
Ciclosporin	-	1 (10)	-
Azathioprine	-	1 (10)	-
Thalidomide	-	1 (10)	-
No medications	10 (35)	2 (20)	-

Normally distributed data are presented as mean±SD, whereas nonparametric data are presented as median , minimal , and maximal values.

N, number; CD, Crohn's disease; UC, ulcerative colitis;

Behaviors: B1, nonstricturing; B2, stricturing, B3, penetrating; p, perianal disease;

Locations: L1, terminal ileum; L2, colon; L3, ileocolon. E1, ulcerative proctitis; E2, left-sided UC (distal to splenic flexure); E3, extensive (proximal to splenic flexure)
