# Supplementary material for: A lncRNA from an inflammatory bowel disease risk locus maintains intestinal host-commensal homeostasis
Source: Cell Res. 2023 Apr 13;33(5):372–88. doi: 10.1038/s41422-023-00790-7 (PMC10156687; doi:10.1038/s41422-023-00790-7)
Supplement: Supplementary file 18 — Supplementary information, Table S3 [file 41422_2023_790_MOESM18_ESM.pdf]

Supplementary information Table 3 Antibody-Reagent-Software

REAGENT or RESOURCE	SOURCE	IDENTIFIER
**Antibodies**		
Pacific Blue CD45.1,clone A20	Biolegend	Cat# 110722
PE CD45RB,clone C363-16A	Biolegend	Cat# 103308
PerCP/Cy5.5 CD4,clone GK1.5	Biolegend	Cat# 100434
FITC CD3,clone 17A2	Biolegend	Cat# 100204
APC CD3,clone 17A2	Biolegend	Cat# 100236
APC CD45.2,clone 104	Biolegend	Cat# 109814
APC CD25,clone PC61	Biolegend	Cat# 102012
IRF-1 (D5E4) XP® Rabbit mAb	CST	Cat#8478
HA-Tag (6E2) Mouse mAb	CST	Cat#2367
Beta Actin Antibody	Proteintech	60009-1-Ig
Lamin B1 Antibody	Proteintech	12987-1-AP
Anti-GFP Rabbit monoclonal antibody	Boster	BM3883
Anti-Histone H3 (acetyl K27) antibody - ChIP Grade	Abcam	Cat#ab4729
Anti-Histone H3 (tri methyl K4) antibody - ChIP Grade	Abcam	Cat#ab8580
Acetyl-Histone H3 (Lys9) (C5B11)	CST	Cat#9649
Anti -CD11b Rabbit pAb	Servicebio	Cat#GB11058
FITC conjugated Goat Anti-Rabbit IgG (H+L)	Servicebio	Cat#GB22303
Cy5 conjugated Goat Anti-Rabbit IgG (H+L)	Servicebio	Cat#GB27303
Cy3 conjugated Goat Anti-Rabbit IgG (H+L)	Servicebio	Cat#GB21303
Anti-HA Magnetic Beads	MedChemExpress	Cat. No.: HY-K0201
**Chemicals, Peptides, and Recombinant Proteins**		
Dextran sulfate sodium salt, colitis grade (36,000 - 50,000)	MP Biomedicals	Cat#160110
TRIzol™ Reagent	Invitrogen	Cat#15596018
TNBS	Sigma	Cat#p2297
Fetal bovine serum	CLARK	FB25015
RPMI-1640	Hyclone	SH30809.01
Dulbecco's Modified Eagle Medium	Hyclone	SH30022.01
Clodronate liposomes	Liposoma BV,Amsterdam,The Netherlands	Cat#CP-010-010
Lipofectamine™ RNAiMAX Transfection Reagent	Invitrogen	Cat#13778075
Human *IRF1* siRNA	GenePharma	
Human *CARING* siRNA	GenePharma	
Human CARING Cy3 FISH Probes	GenePharma	
**Critical Commercial Assays**		
ChIP Assay Kit	Beyotime	P2078
**Software and Algorithms**		
FlowJo	TreeStar	https://www.flowjo.com/
GraphPad Prism version 7	GraphPad Software	https://www.graphpad.com/
R v3.5.3	CRAN	https://r-project.org
Geneious Prime	Geneious	https://www.geneious.com/
Caseviewer software	Caseviewer software (3DHISTECH)	https://www.3dhistech.com/
metascape	metascape	https://metascape.org/
GSEA	GSEA	https://www.gsea-msigdb.org/
usearch(v8.1)	usearch	http://www.drive5.com/usearch/
Qiime(v1.9.1)	Qiime	http://qiime.org/
